# Gene expression profiling of pancreatic ductal adenocarcinomas in response to neoadjuvant chemotherapy

**DOI:** 10.1002/cam4.6411

**Published:** 2023-08-02

**Authors:** Sumit Sahni, Christopher Nahm, Mahsa S. Ahadi, Loretta Sioson, Sooin Byeon, Angela Chou, Sarah Maloney, Elizabeth Moon, Nick Pavlakis, Anthony J. Gill, Jaswinder Samra, Anubhav Mittal

**Affiliations:** ^1^ Northern Clinical School, Faculty of Medicine and Health University of Sydney St Leonards New South Wales Australia; ^2^ Northern Clinical School, Kolling Institute of Medical Research University of Sydney St Leonards New South Wales Australia; ^3^ Australian Pancreatic Centre Sydney New South Wales Australia; ^4^ Western Clinical School, Faculty of Medicine and Health University of Sydney St Leonards New South Wales Australia; ^5^ Department of Anatomical Pathology, NSW Health Pathology Royal North Shore Hospital Sydney New South Wales Australia; ^6^ Northern Sydney Cancer Center, Royal North Shore Hospital St Leonards New South Wales Australia; ^7^ Northern Cancer Institute St Leonards New South Wales Australia; ^8^ Upper Gastrointestinal Surgical Unit Royal North Shore Hospital and North Shore Private Hospital St Leonards New South Wales Australia; ^9^ The University of Notre Dame Australia Sydney New South Wales Australia

**Keywords:** biomarkers, chemotherapy response, gene expression analysis, neoadjuvant chemotherapy, pancreatic ductal adenocarcinoma

## Abstract

**Aim:**

Pancreatic ductal adenocarcinoma (PDAC) has the lowest survival rate of all major cancers. Chemotherapy is the mainstay systemic therapy for PDAC, and chemoresistance is a major clinical problem leading to therapeutic failure. This study aimed to identify key differences in gene expression profile in tumors from chemoresponsive and chemoresistant patients.

**Methods:**

Archived formalin‐fixed paraffin‐embedded tumor tissue samples from patients treated with neoadjuvant chemotherapy were obtained during surgical resection. Specimens were macrodissected and gene expression analysis was performed. Multi‐ and univariate statistical analysis was performed to identify differential gene expression profile of tumors from good (0%–30% residual viable tumor [RVT]) and poor (>30% RVT) chemotherapy‐responders.

**Results:**

Initially, unsupervised multivariate modeling was performed by principal component analysis, which demonstrated a distinct gene expression profile between good‐ and poor‐chemotherapy responders. There were 396 genes that were significantly (*p* < 0.05) downregulated (200 genes) or upregulated (196 genes) in tumors from good responders compared to poor responders. Further supervised multivariate analysis of significant genes by partial least square (PLS) demonstrated a highly distinct gene expression profile between good‐ and poor responders. A gene biomarker of panel (*IL18*, *SPA17*, *CD58*, *PTTG1*, *MTBP*, *ABL1*, *SFRP1*, *CHRDL1*, *IGF1*, and *CFD*) was selected based on PLS model, and univariate regression analysis of individual genes was performed. The identified biomarker panel demonstrated a very high ability to diagnose good‐responding PDAC patients (AUROC: 0.977, sensitivity: 82.4%; specificity: 87.0%).

**Conclusion:**

A distinct tumor biological profile between PDAC patients who either respond or not respond to chemotherapy was identified.

## Graphical abstract

This study aimed to identify underlying differences in the transcriptomic profile of tumors from PC patients with good‐ and poor‐chemotherapy response. A novel gene biomarker signature for chemotherapy response in PC was identified. Moreover, this study also identified oncological pathways and processes that were differentially regulated in tumor tissue based on chemotherapy response and could be developed as putative drug targets to overcome chemoresistance in PC patients.

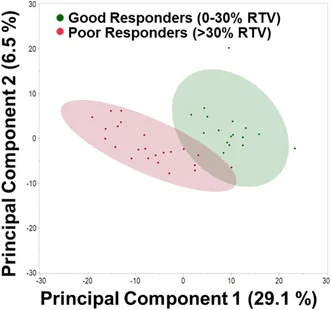

## 1 INTRODUCTION

Pancreatic ductal adenocarcinoma (PDAC) has one of the lowest survival rates, reported to be between 2%–9%.^1^ It is projected to become the second leading cause of cancer‐related mortality by 2030.^2^ Chemotherapy is the mainstay systemic therapeutic option for the majority of PDAC patients.^3^ However, chemotherapy resistance is a major clinical problem in PDAC and results in treatment failure.^3^ There has also been an increase in the use of neoadjuvant chemotherapy (NAC; i.e., pre‐operative chemotherapy) in the treatment regimen of patients who have borderline resectable or locally advanced disease, due to the number of advantages: (1) to achieve more R0 resections with clear margins; (2) for downstaging the disease to enable more patients to undergo curative‐intent resection; (3) treat undetectable micro‐metastatic disease; (4) administer chemotherapy to all PC patients, as a number of patients are too fragile to tolerate chemotherapy after major pancreatic surgery.^4^ However, not all patients respond well to NAC and there is a need to identify key differences in the tumor biology of patients who respond or not respond to NAC.

Gene expression analysis is a gold standard approach to assess the differences in tumor biology. Nanostring nCounter‐based targeted gene expression assays provides a comprehensive coverage of genes associated with cancer progression. Here, we report on the use of Nanostring nCounter‐based discovery approach to identify key differences in tumor biology in patients who respond or not respond to NAC. Furthermore, potential biomarkers for NAC response and putative drug targets to overcome chemoresistance in PDAC patients were identified.

## 2 MATERIALS AND METHODS

### 2.1 Specimen information

Archived formalin‐fixed paraffin‐embedded (FFPE) blocks of PDAC tissue specimens were obtained from the Department of Anatomical Pathology, Royal North Shore Hospital. Specimens from patients treated with NAC between April 2014 and March 2019 at either Royal North Shore Hospital (RNSH) or North Shore Private Hospital (Sydney, Australia) were utilized. The decision to administer NAC was taken after individual discussion by the Pancreatic Cancer Multidisciplinary Team (MDT) at the RNSH. As a standard practice, both upfront resectable and borderline patients are considered for NAC at the RNSH MDT. The NAC regimen was at the discretion of the oncologist. Patients with sufficient tissue available for analysis were included in this study. In addition to NAC patient cohort, a small number of patients treated with upfront surgery were also included as controls. This study was approved by the Northern Sydney Local Health District (NSLHD) Human Research Ethics Committee (Reference# 2019/ETH08639). A waiver of consent was obtained from NSLHD HREC to use archived tissue blocks under NSW Human Tissue Act 1983.

### 2.2 Determination of neoadjuvant chemotherapy response

The response to NAC was determined by the assessment of residual tumor viability by the reporting pathologist, as described previously.^5^, ^6^ A patient with complete regression following NAC treatment was recorded 0% viable tumor, and a patient with no response to NAC was recorded as 100% viable tumor.

### 2.3 RNA extraction

Hematoxylin and eosin (H&E) stained sections of tissue were marked for tumor regions by an experienced pathologist. In order to enrich samples with tumor tissue, FFPE sections (5 μm thickness) were macrodissected based on the marked tumor regions. Total RNA was extracted using Qiagen RNeasy FFPE Kit (Cat# 73504; Qiagen) or Qiagen AllPrep DNA/RNA FFPE Kit (Cat# 80234; Qiagen), following the manufacturer's protocol. RNA quality and quantification were determined by Agilent Bioanalyzer using Agilent RNA 6000 Nano Kit (Agilent Technologies). Samples with at least 25% of fragments greater than 300 nucleotides were used.

### 2.4 Gene expression analysis

Nanostring nCounter PanCancer Progression and nCounter PanCancer Immune Profiling panels (Nanostring Technologies, Seattle) were utilized. In addition, a set of 30 customized gene panel (Table S1) was also added to PanCancer Progression or PanCancer Immune Profile panels. Housekeeping genes were included in the panels for data normalization. RNA samples were processed according to manufacturer's established protocol.^7^ A total of 1366 distinct genes were assessed for their gene expression levels in PDAC tumor specimens.

### 2.5 Data analysis

Analysis of gene counts was performed using nSolver Analysis Software 4.0 (Nanostring Technologies). Normalized data from all three panels were pooled together for further analysis. Principal component analysis (PCA) was performed to determine the inherent groupings within the data. The levels of genes were compared using Multiple *t*‐test with statistical significance achieved at *p* < 0.05. Multivariate analysis was performed using partial least square (PLS) method to identify genes with high diagnostic potential to determine chemotherapy response in patients. Multivariate Receiver Operating Curve was developed using Discriminant Analysis. Survival data were compared using Kaplan–Meier curves and statistically analyzed by the log‐rank test. All statistical analysis was performed using GraphPad Prism 8.4.2 (GraphPad) or JMP Pro 14 (SAS Institute) software. Pathway analysis was performed using nSolver Advanced Analytics. Further, key upstream regulators were predicted using Qiagen Ingenuity Pathway Analysis (Qiagen).

## 3 RESULTS

### 3.1 Patient characteristics

A total of 48 pathologically confirmed PDAC patients (29 males, 19 females) who underwent pancreatic resection surgery were included in the study. 40 patients received neoadjuvant chemotherapy (NAC) and 8 patients underwent upfront surgery. Patient characteristics (age, sex, tumor stage, NAC type, residual tumor viability) are described in Table 1. Initially, patients who underwent NAC treatment were divided into two groups based on their residual tumor viability (RTV), as described previously^5^: (1) good responders (0%–30% RTV) and (2) poor responders (>30% RTV).

**TABLE 1:** Patient characteristics: sex, number of patients, median age, disease stage, type of neoadjuvant therapy, and residual tumor viability of PDAC patients included in this study.

Sex	
Male	29
Female	19
Median age (Years)	67.5
Stage	
IA	1
IB	14
IIA	2
IIB	25
III	2
IV	3
Unknown	1
NAC	
Yes	40
No	8
NAC type	
FOLFIRINOX	14
Gemcitabine/Abraxane	21
Other^a^	2
Unknown	3
Residual tumor viability	
0%–30%	17
>30%	23

Abbreviations: NAC, neoadjuvant chemotherapy; PDAC, pancreatic ductal adenocarcinoma.

^a^ One patient received both FOLFIRINOX and Gemcitabine/Abraxane and one patient received combination of Gemcitabine and Capecitabine.

### 3.2 Gene expression analysis – NAC response biomarkers

**FIGURE 1:**
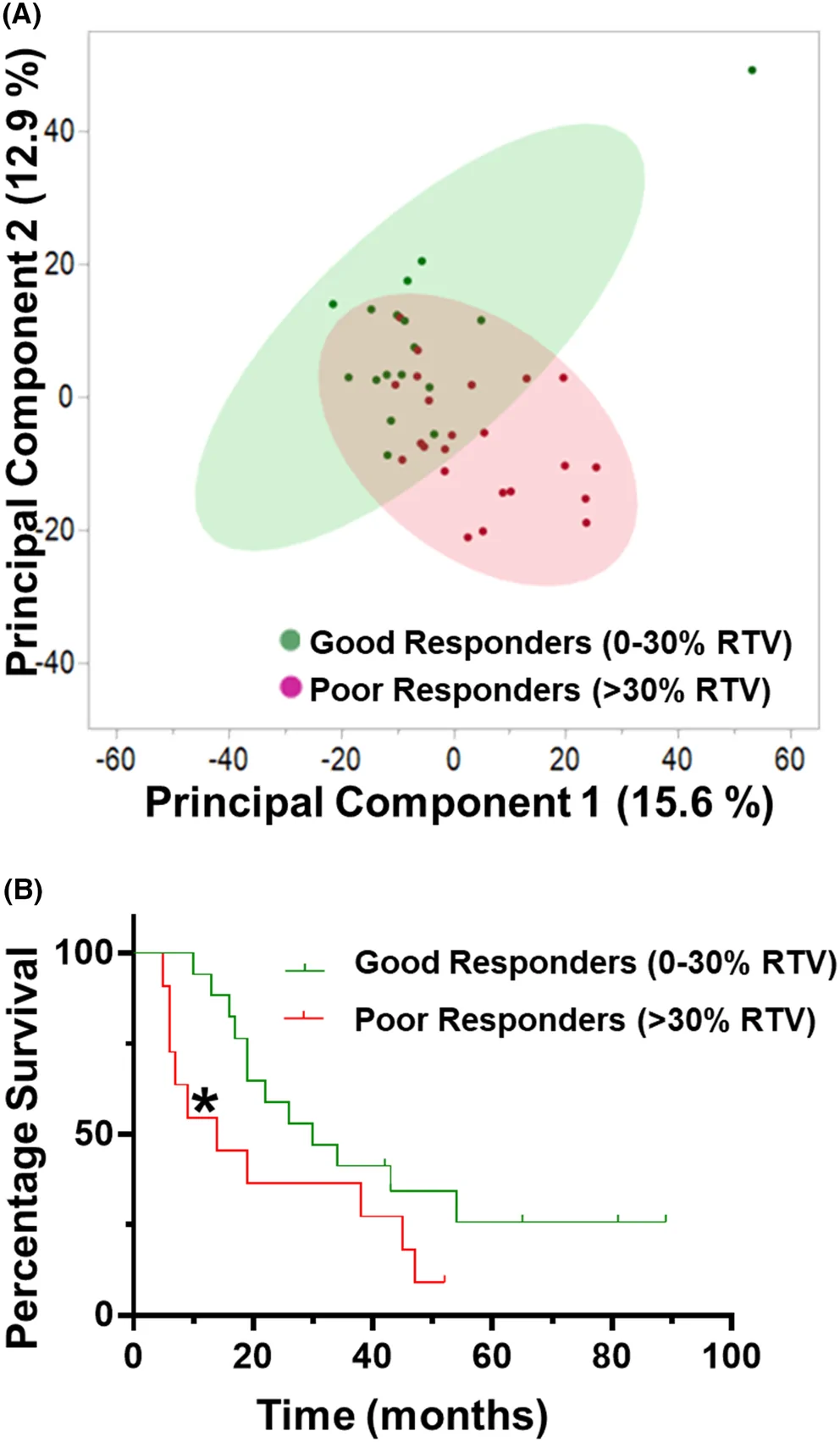
(A) Unsupervised multivariate analysis. Principal component analysis score plot between first two principal components derived from the gene expression profile of tumor tissue obtained from neoadjuvant chemotherapy (NAC)‐treated patients with good‐ (residual tumor viability [RTV]: 0%–30%) and poor‐NAC response (RTV: >30%). (B) Survival analysis based on NAC response. Kaplan–Meier survival curve for patients with either good‐ (RTV: 0%–30%) or poor‐ (RTV: >30%) NAC response. *, *p* < 0.05.

Normalized gene expression data were obtained for PDAC tumor specimens. Principal component analysis was performed on the gene expression data obtained from PDAC patients who received NAC. A distinct gene profile was obtained from patients who had good response to NAC (0%–30% RTV) compared to poor responders (>30% RTV; Figure 1A). Notably, there was a significant (*p < 0.05*) survival difference in patients classified as good‐ and poor‐NAC responders (Figure 1B).

There was a total of 396 significantly (*p* < 0.05) different genes in tumors from PDAC patients with good‐NAC response compared to poor responders (Table S2). Of these, 200 genes were significantly increased and 196 were significantly decreased in good‐NAC responders compared to poor responders. The top 10 significantly increased or decreased genes are reported in Table 2.

**TABLE 2:** List of top 10 significant genes with increased or decreased expression in good‐NAC responders compared to poor‐NAC responders.

Gene	*p*‐Value	Good responders	Poor responders	Fold change
Increased expression				
*ACTG2*	0.023	5885	496	11.86
*MYH11*	0.021	5617	660	8.51
*CNN1*	0.012	817	113	7.23
*PTX3*	0.016	1357	240	5.65
*MYLK*	0.017	7784	2015	3.86
*OGN*	0.001	1558	418	3.73
*SFRP1*	0.001	466	126	3.70
*TPM2*	0.01	10,518	3042	3.46
*ADAMTS8*	0.001	147	43.6	3.37
*IGF1*	0.001	687	224	3.07
Decreased expression				
*CT45A1*	0.026	23.4	116	−4.96
*IFNA7*	0.033	23.4	116	−4.96
*MUC16*	0.024	66.5	261	−3.92
*ULBP2*	0.004	24.4	88.7	−3.64
*LAMA3*	0.006	157	504	−3.21
*ITGB6*	0.044	160	513	−3.21
*IFNL2*	0.009	23.6	75.6	−3.20
*LAMB3*	0.007	346	1093	−3.16
*CCL18*	0.028	215	661	−3.07
*TACSTD2*	0.002	1417	4325	−3.05

Abbreviation: NAC, neoadjuvant chemotherapy.

**FIGURE 2:**
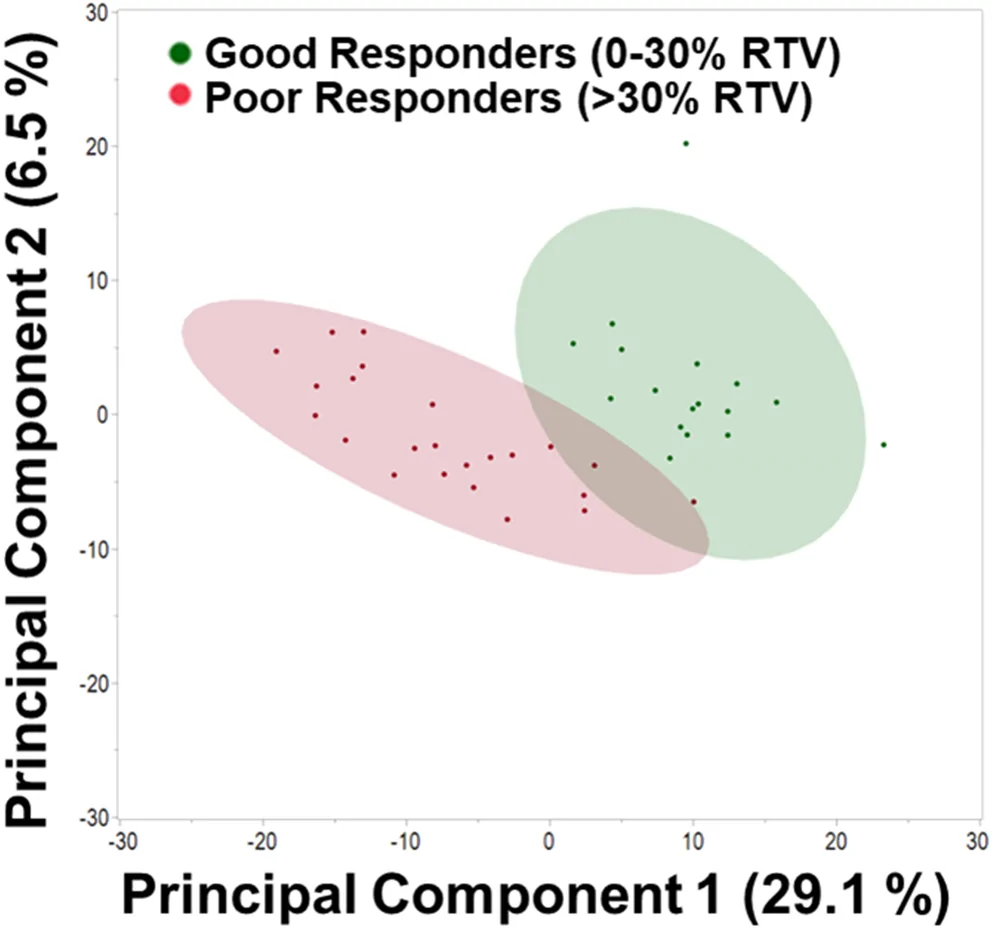
Supervised multivariate analysis. Partial least square score plot between first two components derived from the significantly different genes between tumor tissue obtained from neoadjuvant chemotherapy‐treated patients with good response (residual tumor viability [RTV]: 0%–30%) and poor response (RTV: >30%).

**FIGURE 3:**
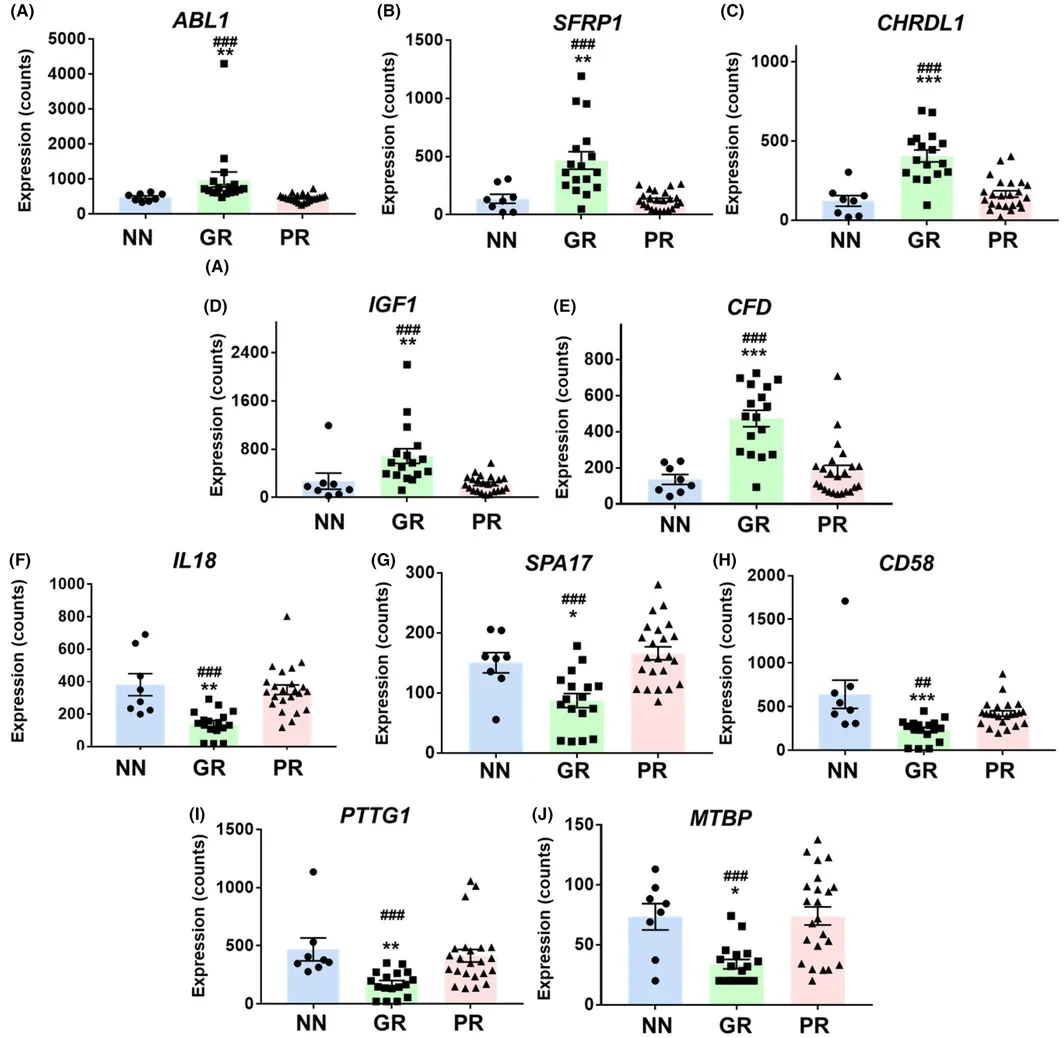
Gene expression levels of identified biomarkers. Gene expression levels of 10 identified biomarkers was compared between patients with good‐neoadjuvant chemotherapy (NAC) response (GR), poor‐NAC response (PR) and patients who underwent upfront surgery (i.e., No‐NAC [NN]). (A) *ABL1*; (B) *SFRP1*; (C) *CHRDL1*; (D) *IGF1*; (E) *CFD*; (F) *IL18*; (G) *SPA17*; (H) *CD58*; (I) *PTTG1*; and (J) *MTBP*. *, *p* < 0.05; **, *p* < 0.01; ***, *p* < 0.001 compared to NN; ^#^, *p* < 0.05; ^##^, *p* < 0.01; ^###^, *p* < 0.001 compared to PR.

To further identify key discriminatory genes between good‐ and poor‐NAC responders, multivariate PLS model was generated using data from significantly different genes. A distinct class grouping (i.e., good responders vs. poor responders) was observed in PLS model (Figure 2). Most influential genes were selected based on the Variable Importance in Projection (VIP) score (cut‐off >1.0) and AUROC values obtained from univariate logistic regression (Table S3). The top 5 genes with increased (*ABL1*, *SFRP1*, *CHRDL1*, *IGF1*, and *CFD*) and decreased (i.e., *IL18*, *SPA17*, *CD58*, *PTTG1*, and *MTBP*) expression levels (Table S3), in good responders compared to poor responders, were selected for developing a biomarker panel, which can distinguish PDAC tumors based on the NAC response. Upon univariate analysis, the levels of 10 biomarkers were significantly (*p* < 0.05) different for good‐NAC responders compared to poor‐NAC responders or patients who received upfront surgery (i.e., No‐ NAC cohort; Figure 3). Further, this unique biomarker panel was used to develop a multivariate discriminant analysis model and demonstrated an AUROC of 0.977 to diagnose patients with good‐NAC response compared to poor responders (sensitivity: 82.4% and specificity: 87.0%).

### 3.3 Gene expression analysis – Prognostic biomarkers

**FIGURE 4:**
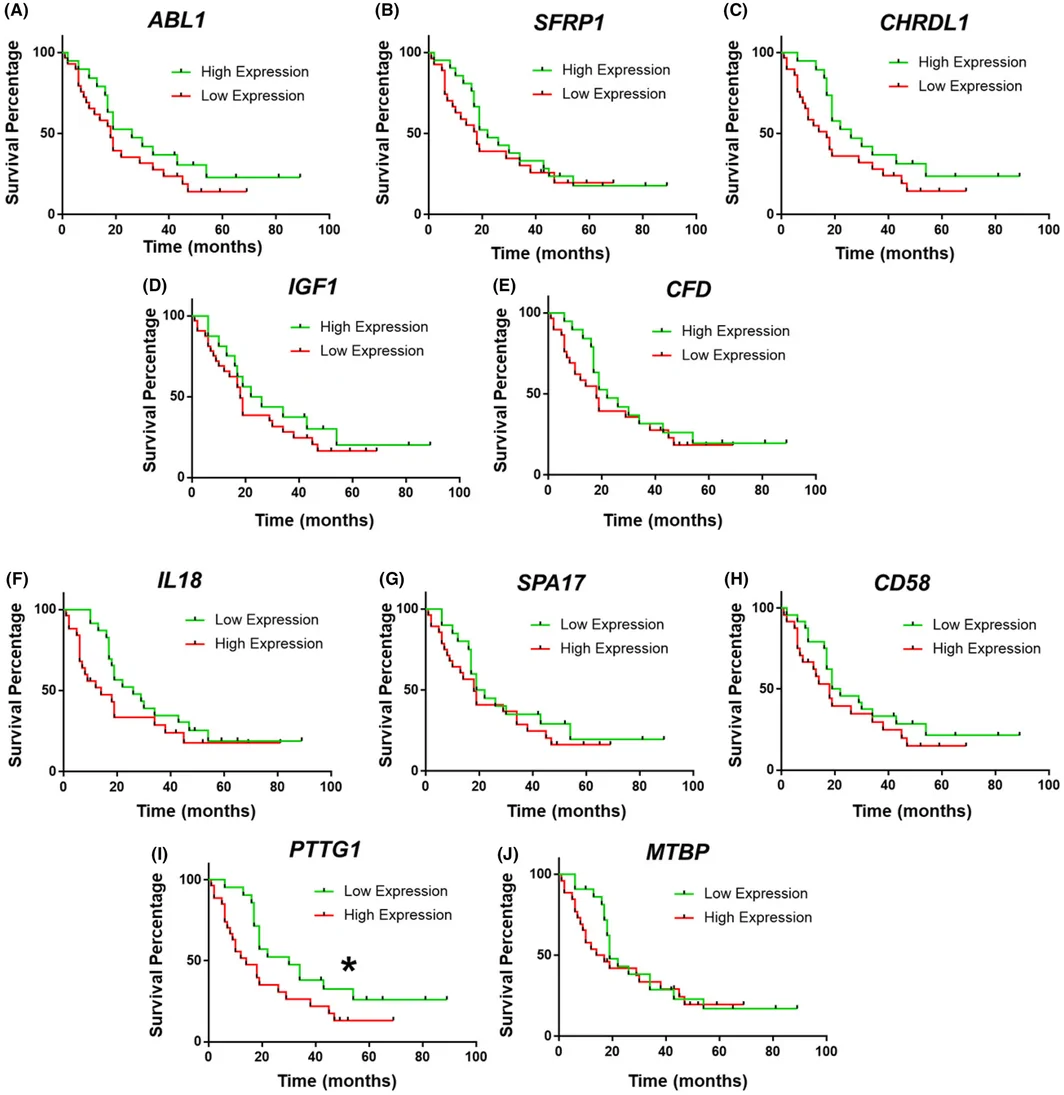
Survival analysis of identified biomarkers. Kaplan–Meier survival curves of 10 identified biomarkers based on their gene expression levels. (A) *ABL1*; (B) *SFRP1*; (C) *CHRDL1*; (D) *IGF1*; (E) *CFD*; (F) *IL18*; (G) *SPA17*; (H) *CD58*; (I) *PTTG1*; and (J) *MTBP*. *, *p* < 0.05.

Finally, we determined the prognostic (i.e., overall survival [OS]) significance of the identified biomarker panel. The patients were divided into high and low expression levels with cut‐off (Table S4) based on the highest Youden Index (i.e., Sensitivity + Specificity – 1). Only expression level of *PTTG1* gene was shown to have significant prognostic effect, with decreased levels in patients resulting in significantly (*p* < 0.05) good prognostic outcomes (Median OS: 30 months) compared to patients with increased levels (median OS: 14 months; Figure 4). After multivariable analysis, poor prognosis (hazard ratio: 1.802) was associated with expression level of *PTTG1* gene, but this effect was not significant (*p* = 0.12; Table S5).

### 3.4 Pathway analysis and potential upstream regulators

**FIGURE 5:**
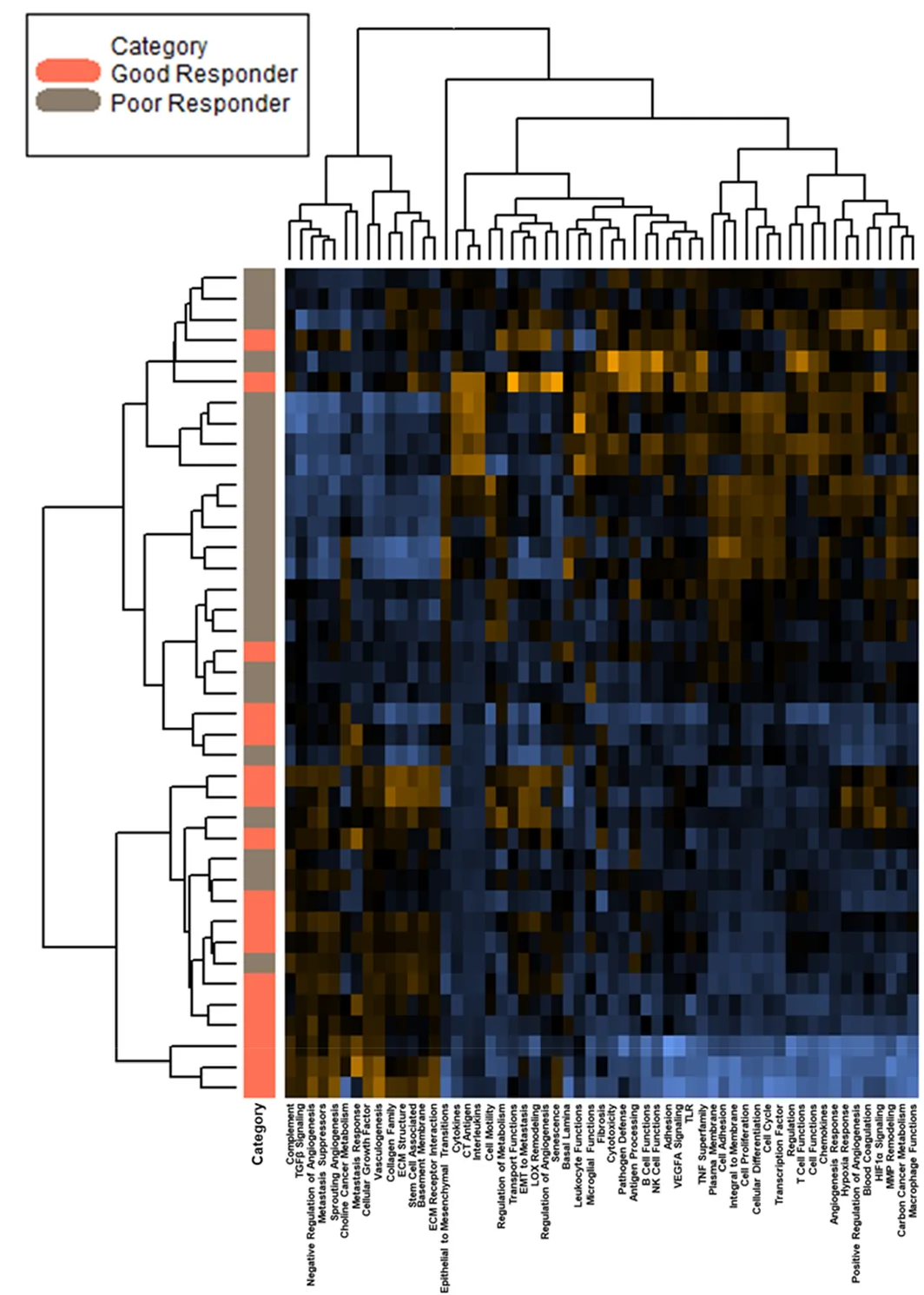
Pathway analysis. Heatmap of differentially regulated pathways between good‐neoadjuvant chemotherapy (NAC) responders compared to poor‐NAC responders. Orange indicates high scores; blue indicates low scores. Scores are displayed on the same scale via a Z‐transformation.

Pathway analysis based on the gene expression profile demonstrated distinct pathways involved in the tumors from good‐ and poor‐NAC responders. The good‐NAC responders had increased activity in pathways related to metastasis suppression, ECM structure, etc., while there was a decrease in the activity of pathways related to immune response, metastasis progression, angiogenesis, etc. (Figure 5). However, there was no significant changes observed in the levels of immune cells between good‐ and poor‐NAC responders (Figure S1). Finally, Ingenuity Pathway Analysis was further used to identify potential upstream regulators (*z* score >2.0; *p*‐value of overlap <0.05) that could be responsible for the observed gene expression profile (Table S5).

## 4 DISCUSSION

PDAC patients have limited systemic therapeutic options, with conventional chemotherapy being majorly used for the treatment.^3^ However, chemotherapy does not provide a curative‐intent treatment option, primarily due to high prevalence of chemoresistance in pancreatic tumors.^3^ Herein, using tumor specimens from NAC‐treated PDAC patients, differential gene expression profile was assessed between patients who have either responded or not responded to NAC. A group of genes were identified as a potential biomarker panel for chemoresponse in PDAC patients. Furthermore, pathway analysis identified key differences in the oncogenic signaling pathways based on chemoresponse. Key upstream regulators of the observed differential gene expression profile were also identified, which could be developed as targets to overcome chemoresistance in PDAC.

Currently, surgery is the only curative‐intent treatment option for PDAC patients.^3^ The patients who are candidates for surgery are treated with multimodal treatment with chemotherapy before (i.e., NAC) and after (i.e., adjuvant chemotherapy) surgical resection. Chemotherapy response under neoadjuvant settings could potentially act as a predictor for response under adjuvant settings. Hence, the identified biomarker panel for NAC response in this study could be used to identify patients who need to be monitored closely for disease relapse during their adjuvant treatment. In addition, future validation of these biomarkers on pre‐NAC diagnostic biopsies could open the door for a predictive biomarker signature for NAC response. This will be highly beneficial for clinical decision‐making around the treatment course for an individual patient, especially in patients presenting with upfront resectable disease.

Biomarker panel with increased levels of *ABL1*, *SFRP1*, *CHRDL1*, *IGF1*, and *CFD*, and decreased levels of *IL18*, *SPA17*, *CD58*, *PTTG1*, and *MTBP* was able to identify patients with good‐NAC response with high sensitivity and selectivity. Response to chemotherapy is mediated by complex mechanisms involving an array of oncogenic signaling pathways. Hence, a biomarker panel could provide a reflection of complex integrated signaling involved in the observed pattern of chemoresponse in PDAC. In direct concordance with this study, PDAC patients with chemoresistant tumors were previously shown to have increased serum levels of IL18,^8^ indicating that this cytokine could play a role in mediating chemoresistance in PDAC. Similar to our study, lysates from chemosensitive breast tumors had lower IL18 protein levels compared to chemoresistant tumors.^9^ IL18 is previously known to activate NF‐κB pathway,^10^ which was also observed to be a significantly activated upstream regulator in our pathway analysis. Notably, NF‐κB pathway is involved in regulation of multiple oncogenic signaling pathways, which mediate chemoresistance,^11^ and this could be one of the potential mechanisms via which IL18 is involved in mediating chemoresistance in PDAC.

SFRP1 is a negative regulator of Wnt signaling pathway.^12^ Notably, Wnt signaling is known to play a pivotal role in mediating chemoresistance in PDAC,^13^ which could explain the observed increase in *SFRP1* in tumor tissue from good‐NAC responders. Similar to our results, SFRP1 has previously demonstrated to have a positive correlation with chemotherapy response in triple‐negative breast cancer patients treated with NAC.^14^ However, this role of SFRP1 in mediating chemosensitivity in breast cancer was shown to be independent of its effect on Wnt signaling.^14^ Hence, future studies are required to further confirm if the observed increase in SFRP1 in good‐NAC responders in PDAC is dependent or independent of its effect on Wnt signaling pathway.

*CHRDL1* was also shown to be increased in good‐NAC responders and is known to be a negative regulator of BMP4.^15^ Of interest, BMP4 has been previously known to play a role in chemoresistance via regulation of MAPK signaling and apoptosis‐autophagy axis.^16^, ^17^ Interestingly, *CFD* has been recently identified as a marker for a unique subtype of cancer‐associated fibroblasts, namely, complement‐secreting CAFs (csCAFs).^18^ These CAFs are suggested to play a tumor suppressive role^18^ and observed increased levels of *CFD* in macrodissected tumors from good‐responding PDAC patients indicate its potential role in increasing the susceptibility of cancer cells to chemotherapy.

In direct alignment with our results, previous studies have shown that overexpression of SPA17 leads to increased chemoresistance in ovarian cancer cell, while knockdown of SPA17 increased chemosensitivity.^19^, ^20^ However, the exact mechanism via which SPA17 mediate chemoresistance is still elusive. Moreover, CD58 is previously shown to be involved in self‐renewal ability of cancer cells,^21^ and this could be a potential mechanism via which it mediates chemoresistance in PDAC. Similarly, PTTG1 has been previously implicated in regulation of cancer stemness in ovarian cancer.^22^ Cancer stemness is an important mechanism for mediating chemoresistance,^23^ and future studies should focus on establishing the role of PTTG1 in regulating stemness in PDAC. *MTBP1* was another oncogene found to be increased in poor‐NAC responders. MTBP1 is known to interact with MYC to enhance its oncogenic activity.^24^ MYC is known to be involved in chemoresistance in PDAC,^25^ and could be a potential mechanism by which MTBP1 is involved in PDAC chemoresistance.

In contrast to our findings, stroma‐derived IGF1 was previously shown to derive chemotherapy resistance in pancreatic cancer^26^ and requires further analysis using spatial transcriptomics to define precise role of this growth factor in chemoresistance in PDAC. Although mutant ABL1 is known to have oncogenic role, the normal ABL1 was shown to have tumor suppressor function in chronic myeloid leukemia model.^27^ The role of ABL1 in chemoresponse is still elusive and require further mechanistic investigations.

A number of these genes have been shown to be biomarker for poor prognosis in various cancer types, but their ability to discriminate patients based on chemotherapy response is unknown. *SFRP1* is a tumor suppressor gene with decreased levels associated with poor prognostic outcomes in different cancer types including pancreatic cancer.^28^, ^29^, ^30^, ^31^ Similarly, *CHRDL1* is shown to be associated with good prognostic outcomes.^15^, ^32^ Increased levels of SPA17 and CD58 were previously linked to poor prognostic outcome in breast and pancreatic cancer patients, respectively.^33^, ^34^ Similarly, higher levels of MTBP are also associated with poor prognostic outcomes in various cancer types.^35^, ^36^, ^37^ Although there were some differences in survival curves observed based on the levels of these abovementioned genes in the current study, they did not achieve statistically significant prognostic significance, which could be due to a relatively smaller cohort size.

Notably, tissue levels of *PTTG1* gene were able to stratify patients into two groups based on their overall survival, with patients with high expression levels resulting in poor prognostic outcome (i.e., overall survival). This gene encodes for PTTG1, which is an oncogenic transcription factor known to be upregulated in number of malignancies.^38^ PTTG1 is involved in promotion of angiogenesis via FGF2 and VEGF and cellular proliferation.^39^, ^40^

Pancreatic resection is a highly morbid procedure and biomarkers are required to determine if a patient will benefit from it. Understanding the differences in tumor biology of patients who respond or not respond to chemotherapy is of critical importance in attempt to develop novel therapeutic modalities in future. The pathway analysis of gene expression data demonstrated a decrease in activity of pathways associated with cancer metastasis, angiogenesis and immune system in good‐NAC responders. This latter observation was in contrast with our previous proteomic study,^5^ where mass spectrometry‐based analysis of bulk tumor demonstrated heightened immune response pathways in good‐NAC responders. These results highlight the importance of spatial heterogeneity that exists in the tumor microenvironment and future spatial transcriptomic analysis will be required to better understand the role of immune pathways in chemotherapy response in PDAC patients.

Notably, there are no targeted treatment options available for the majority of PDAC patients. Hence, identifying new putative targets to develop novel targeted therapies is critical. A number of key upstream regulators were identified in this study, which could be targeted to overcome chemoresistance. Interestingly, *PKRAA* (gene encoding AMPK) was one of the key upstream regulators identified. AMPK is a key energy homeostasis regulator and could provide plasticity to tumors to overcome hostile conditions in the tumor microenvironment as well as due to treatment with chemotherapy.^41^, ^42^ AMPK also activates autophagy, which is an intrinsic cell survival pathway and is known to play an important role in PDAC progression.^43^ Hence, targeting AMPK pathway could be a novel therapeutic avenue to overcome chemoresistance in PDAC and future studies validating and targeting this kinase to overcome chemoresistance will be of interest.

The main limitations of this study are (1) retrospective study design, (2) relatively small sample size, and (3) use of macrodissected specimens. Future multicenter prospective study with a larger patient cohort will be required to further validate these findings. A larger cohort will further allow to perform NAC regimen‐specific analysis, which was currently not possible due to relatively small number of patients in each cohort. Moreover, use of spatial profiling, instead of macrodissection, will provide further deeper understanding of these differences in tumor biology in PDAC in response to chemotherapy treatment.

## 5 CONCLUSION

Overall, this study has demonstrated key differences in the gene expression profile of patients who respond to chemotherapy compared to nonresponders. A distinct tumor biological profile was identified in PDAC patients with NAC‐responsive tumor phenotype, which provides further insight into potential underlying mechanisms responsible for marked variability in clinical activity of chemotherapy against pancreatic tumors. Further research is required to validate these novel findings.

## AUTHOR CONTRIBUTIONS

**Sumit Sahni:** Conceptualization (equal); data curation (equal); formal analysis (equal); funding acquisition (equal); methodology (equal); project administration (equal); resources (equal); writing – original draft (equal); writing – review and editing (equal). **Christopher Nahm:** Methodology (equal); resources (equal); writing – review and editing (equal). **Mahsa S. Ahadi:** Investigation (equal); methodology (equal); writing – review and editing (equal). **Loretta Sioson:** Methodology (equal); writing – review and editing (equal). **Sooin Byeon:** Data curation (equal); methodology (equal); writing – review and editing (equal). **Angela Chou:** Methodology (equal); writing – review and editing (equal). **Sarah Maloney:** Data curation (equal); writing – review and editing (equal). **Elizabeth Moon:** Methodology (equal); writing – review and editing (equal). **Nick Pavlakis:** Resources (equal); supervision (equal); writing – review and editing (equal). **Anthony J. Gill:** Data curation (equal); methodology (equal); resources (equal); writing – review and editing (equal). **Jaswinder Samra:** Resources (equal); supervision (equal); writing – review and editing (equal). **Anubhav Mittal:** Conceptualization (equal); funding acquisition (equal); project administration (equal); resources (equal); supervision (equal); writing – review and editing (equal).

## FUNDING INFORMATION

This study was supported by a Project Grant and an Excellence in Pancreatic Cancer Grant from the Sydney Vital Translational Cancer Research Centre.

## CONFLICT OF INTEREST STATEMENT

No conflicts of interest were declared by the authors.

## ETHICS STATEMENT

This study was approved by the Northern Sydney Local Health District (NSLHD) Human Research Ethics Committee (Reference# 2019/ETH08639).

## CONSENT

A waiver of consent was obtained from NSLHD HREC to use archived tissue blocks under NSW Human Tissue Act 1983.

## Supporting information


Data S1:

## Data Availability

The data supporting the findings of this study are available from the corresponding author upon reasonable request.
