# Supplementary material for: Gene expression profiling of pancreatic ductal adenocarcinomas in response to neoadjuvant chemotherapy
Source: Cancer Med. 2023 Aug 2;12(17):18050–61. doi: 10.1002/cam4.6411 (PMC10523964; doi:10.1002/cam4.6411)
Supplement: Supplementary file 1 — Data S1: [file CAM4-12-18050-s001.docx]

| **S.No.** | **Gene Name** | **Accession #** | **Position** |
| --- | --- | --- | --- |
| **1** | *ABCB1* | NM_000927.3 | 3911-4010 |
| **2** | *BECN1* | NM_003766.2 | 811-910 |
| **3** | *CA11* | NM_001217.3 | 1128-1227 |
| **4** | *CD274* | NM_014143.3 | 50-149 |
| **5** | *CD276* | NM_001024736.1 | 2121-2220 |
| **6** | *CTLA4* | NM_005214.3 | 406-505 |
| **7** | *DCK* | NM_000788.2 | 311-410 |
| **8** | *EPHA3* | NM_005233.5 | 1280-1379 |
| **9** | *FSCN1* | NM_003088.2 | 1844-1943 |
| **10** | *HSPA5* | NM_005347.3 | 3636-3735 |
| **11** | *IDO1* | NM_002164.5 | 370-469 |
| **12** | *ITIH4* | NM_002218.3 | 1746-1845 |
| **13** | *ITPRIP* | NM_033397.2 | 4146-4245 |
| **14** | *MAP1LC3B* | NM_022818.4 | 1686-1785 |
| **15** | *MSLN* | NM_013404.3 | 1179-1278 |
| **16** | *MUC16* | NM_024690.2 | 43456-43555 |
| **17** | *PDCD1LG2* | NM_025239.3 | 236-335 |
| **18** | *PDE8B* | NM_003719.2 | 1896-1995 |
| **19** | *PGAM1* | NM_002629.2 | 1038-1137 |
| **20** | *PRKAA1* | NM_006251.5 | 367-466 |
| **21** | *PRKAA2* | NM_006252.2 | 976-1075 |
| **22** | *PTGES2* | NM_025072.6 | 1713-1812 |
| **23** | *RACGAP1* | NM_013277.3 | 1851-1950 |
| **24** | *RASGRF2* | NM_006909.2 | 626-725 |
| **25** | *RBP1* | NM_002899.3 | 446-545 |
| **26** | *S100A2* | NM_005978.3 | 568-667 |
| **27** | *S100A4* | NM_002961.2 | 264-363 |
| **28** | *SLC29A1* | NM_001078177.1 | 1726-1825 |
| **29** | *SQSTM1* | NM_003900.3 | 1446-1545 |
| **30** | *TMPO* | NM_001032284.1 | 577-676 |

**Table S1:** List of additional genes added to the Nanostring nCounter Panels.

**Table S2:** List of genes with significant differential expression between tumours from good- and poor-NAC responders.

| **Gene** | ***p* value** | **Good Responders** | **Poor Responders** | **Fold Change** |
| --- | --- | --- | --- | --- |
| *ACTG2* | 0.023 | 5885 | 496 | 11.86 |
| *MYH11* | 0.021 | 5617 | 660 | 8.51 |
| *CNN1* | 0.012 | 817 | 113 | 7.23 |
| *PTX3* | 0.016 | 1357 | 240 | 5.65 |
| *MYLK* | 0.017 | 7784 | 2015 | 3.86 |
| *OGN* | 0.001 | 1558 | 418 | 3.73 |
| *SFRP1* | 0.001 | 466 | 126 | 3.70 |
| *TPM2* | 0.01 | 10518 | 3042 | 3.46 |
| *ADAMTS8* | 0.001 | 147 | 43.6 | 3.37 |
| *IGF1* | 0.001 | 687 | 224 | 3.07 |
| *C7* | 0.012 | 8128 | 2779 | 2.92 |
| *THBS4* | 0.003 | 1401 | 519 | 2.70 |
| *CSPG4* | 0.001 | 515 | 195 | 2.64 |
| *TNXB* | 0.001 | 388 | 148 | 2.62 |
| *CFD* | 0.001 | 473 | 182 | 2.60 |
| *ITGA7* | 0.001 | 275 | 107 | 2.57 |
| *FOS* | 0.002 | 12242 | 4836 | 2.53 |
| *C4B* | 0.008 | 4166 | 1668 | 2.50 |
| *FHL1* | 0.001 | 1269 | 512 | 2.48 |
| *CHRDL1* | 0.001 | 406 | 166 | 2.45 |
| *THBS1* | 0.021 | 13822 | 5804 | 2.38 |
| *CCL14* | 0.002 | 320 | 135 | 2.37 |
| *CXCL12* | 0.001 | 1098 | 466 | 2.36 |
| *NR4A1* | 0.011 | 2091 | 920 | 2.27 |
| *F13A1* | 0.007 | 3000 | 1321 | 2.27 |
| *FGF2* | 0.001 | 374 | 166 | 2.25 |
| *CLU* | 0.033 | 6034 | 2682 | 2.25 |
| *ADAMTS1* | 0.001 | 1885 | 846 | 2.23 |
| *SERPINE1* | 0.03 | 5954 | 2701 | 2.20 |
| *CCDC80* | 0.001 | 6449 | 2927 | 2.20 |
| *FBLN5* | 0.001 | 1352 | 616 | 2.19 |
| *FYN* | 0.027 | 1295 | 592 | 2.19 |
| *FEZ1* | 0.024 | 580 | 267 | 2.17 |
| *LTBP4* | 0.001 | 1958 | 905 | 2.16 |
| *ABL1* | 0.008 | 984 | 457 | 2.15 |
| *DPT* | 0.003 | 1225 | 570 | 2.15 |
| *ELK1* | 0.024 | 606 | 287 | 2.11 |
| *IGFBP4* | 0.001 | 10234 | 4868 | 2.10 |
| *FBLN1* | 0.001 | 5695 | 2724 | 2.09 |
| *BAI3* | 0.043 | 93.7 | 44.9 | 2.09 |
| *MFAP4* | 0.001 | 3267 | 1570 | 2.08 |
| *CYP1B1* | 0.005 | 688 | 338 | 2.04 |
| *ABI3BP* | 0.001 | 1277 | 634 | 2.01 |
| *TNC* | 0.036 | 487 | 243 | 2.00 |
| *EGR1* | 0.01 | 1336 | 667 | 2.00 |
| *BCL6* | 0.001 | 1032 | 521 | 1.98 |
| *CFI* | 0.021 | 1046 | 534 | 1.96 |
| *CR2* | 0.012 | 47.4 | 24.3 | 1.95 |
| *TNS1* | 0.001 | 2320 | 1190 | 1.95 |
| *NR4A3* | 0.047 | 625 | 323 | 1.93 |
| *ZCCHC24* | 0.001 | 639 | 333 | 1.92 |
| *STAB1* | 0.003 | 373 | 195 | 1.91 |
| *CDKN1A* | 0.004 | 1966 | 1032 | 1.91 |
| *EMILIN1* | 0.001 | 2164 | 1141 | 1.90 |
| *JAM2* | 0.001 | 269 | 143 | 1.88 |
| *MEG3* | 0.001 | 930 | 501 | 1.86 |
| *GPR124* | 0.002 | 326 | 177 | 1.84 |
| *RGCC* | 0.011 | 2516 | 1370 | 1.84 |
| *PTGIS* | 0.001 | 1090 | 599 | 1.82 |
| *SPARCL1* | 0.001 | 2576 | 1430 | 1.80 |
| *FCER1A* | 0.049 | 208 | 116 | 1.79 |
| *MCAM* | 0.002 | 385 | 215 | 1.79 |
| *BNC2* | 0.003 | 202 | 113 | 1.79 |
| *CD99* | 0.001 | 6818 | 3818 | 1.79 |
| *COL3A1* | 0.019 | 72905 | 40941 | 1.78 |
| *BAG2* | 0.001 | 520 | 294 | 1.77 |
| *LIFR* | 0.003 | 335 | 191 | 1.75 |
| *IL6ST* | 0.003 | 4548 | 2598 | 1.75 |
| *C1R* | 0.006 | 6690 | 3844 | 1.74 |
| *COL6A2* | 0.001 | 6185 | 3556 | 1.74 |
| *FXYD6* | 0.01 | 895 | 516 | 1.73 |
| *TNFSF14* | 0.013 | 163 | 94.4 | 1.73 |
| *CRISPLD2* | 0.001 | 2144 | 1244 | 1.72 |
| *COL6A1* | 0.001 | 3524 | 2054 | 1.72 |
| *RAMP1* | 0.002 | 504 | 297 | 1.70 |
| *SLIT2* | 0.004 | 275 | 164 | 1.68 |
| *MAPKAPK2* | 0.008 | 1104 | 659 | 1.68 |
| *PDPN* | 0.044 | 961 | 574 | 1.67 |
| *STAT3* | 0.002 | 6263 | 3746 | 1.67 |
| *TEK* | 0.001 | 70.9 | 42.5 | 1.67 |
| *CLEC3B* | 0.021 | 172 | 104 | 1.65 |
| *DCN* | 0.024 | 10738 | 6494 | 1.65 |
| *FSTL1* | 0.001 | 7298 | 4428 | 1.65 |
| *ROBO4* | 0.001 | 120 | 72.9 | 1.65 |
| *PLA2G6* | 0.001 | 311 | 189 | 1.65 |
| *MPDZ* | 0.001 | 350 | 213 | 1.64 |
| *PCOLCE* | 0.006 | 1557 | 956 | 1.63 |
| *DLC1* | 0.001 | 302 | 186 | 1.62 |
| *ZEB2* | 0.002 | 500 | 308 | 1.62 |
| *MTA1* | 0.026 | 456 | 281 | 1.62 |
| *ID4* | 0.001 | 588 | 364 | 1.62 |
| *ZEB1* | 0.001 | 717 | 444 | 1.61 |
| *FERMT2* | 0.001 | 644 | 400 | 1.61 |
| *C1S* | 0.012 | 3257 | 2024 | 1.61 |
| *NOS3* | 0.003 | 138 | 86.6 | 1.59 |
| *MAPK14* | 0.022 | 981 | 618 | 1.59 |
| *CD81* | 0.001 | 9908 | 6249 | 1.59 |
| *TWIST2* | 0.026 | 123 | 77.6 | 1.59 |
| *THY1* | 0.018 | 2378 | 1512 | 1.57 |
| *SERPINF1* | 0.025 | 5762 | 3670 | 1.57 |
| *WWTR1* | 0.001 | 1498 | 962 | 1.56 |
| *DPYSL3* | 0.005 | 2434 | 1569 | 1.55 |
| *LRP1* | 0.017 | 2172 | 1406 | 1.54 |
| *CD34* | 0.001 | 281 | 182 | 1.54 |
| *SNAI1* | 0.043 | 301 | 195 | 1.54 |
| *JUN* | 0.025 | 1040 | 675 | 1.54 |
| *RPS6* | 0.004 | 29421 | 19108 | 1.54 |
| *MGP* | 0.014 | 8967 | 5838 | 1.54 |
| *AKAP2* | 0.001 | 648 | 424 | 1.53 |
| *SRF* | 0.001 | 948 | 622 | 1.52 |
| *PECAM1* | 0.005 | 1595 | 1047 | 1.52 |
| *COL1A1* | 0.037 | 55955 | 36738 | 1.52 |
| *JAM3* | 0.005 | 625 | 411 | 1.52 |
| *NRP1* | 0.001 | 2324 | 1533 | 1.52 |
| *AKAP12* | 0.008 | 1219 | 805 | 1.51 |
| *AGGF1* | 0.013 | 130 | 86.2 | 1.51 |
| *SYNE1* | 0.001 | 216 | 144 | 1.50 |
| *NPR1* | 0.024 | 135 | 90.1 | 1.50 |
| *EGFL7* | 0.011 | 360 | 241 | 1.49 |
| *IGF1R* | 0.04 | 1120 | 751 | 1.49 |
| *SPARC* | 0.017 | 17016 | 11414 | 1.49 |
| *FMOD* | 0.001 | 2605 | 1749 | 1.49 |
| *ENTPD1* | 0.008 | 456 | 307 | 1.49 |
| *MAF* | 0.027 | 681 | 461 | 1.48 |
| *PDCL3* | 0.001 | 279 | 189 | 1.48 |
| *CALD1* | 0.009 | 6289 | 4262 | 1.48 |
| *BCL2* | 0.037 | 249 | 169 | 1.47 |
| *ECSCR* | 0.019 | 126 | 85.7 | 1.47 |
| *RORA* | 0.009 | 599 | 409 | 1.46 |
| *ITM2A* | 0.035 | 260 | 178 | 1.46 |
| *PMP22* | 0.03 | 1119 | 767 | 1.46 |
| *PIK3R1* | 0.012 | 970 | 665 | 1.46 |
| *CAMK2D* | 0.001 | 1125 | 774 | 1.45 |
| *SMAD9* | 0.03 | 165 | 114 | 1.45 |
| *GSN* | 0.005 | 5423 | 3759 | 1.44 |
| *PDE8B* | 0.019 | 37 | 25.7 | 1.44 |
| *NFKBIA* | 0.034 | 3405 | 2367 | 1.44 |
| *EPAS1* | 0.003 | 777 | 541 | 1.44 |
| *LHFP* | 0.011 | 904 | 631 | 1.43 |
| *DDR2* | 0.009 | 650 | 455 | 1.43 |
| *HSPG2* | 0.001 | 4747 | 3326 | 1.43 |
| *TXNIP* | 0.015 | 11705 | 8225 | 1.42 |
| *ITGA9* | 0.048 | 172 | 121 | 1.42 |
| *HOXB3* | 0.046 | 604 | 425 | 1.42 |
| *PLEKHO1* | 0.014 | 1016 | 716 | 1.42 |
| *FGFR1* | 0.014 | 1024 | 722 | 1.42 |
| *LAMC1* | 0.001 | 1471 | 1039 | 1.42 |
| *CMA1* | 0.027 | 28.4 | 20.1 | 1.41 |
| *VIM* | 0.009 | 17835 | 12667 | 1.41 |
| *SPHK2* | 0.001 | 312 | 222 | 1.41 |
| *MEF2C* | 0.012 | 441 | 315 | 1.40 |
| *EMP3* | 0.011 | 527 | 380 | 1.39 |
| *MYC* | 0.034 | 1323 | 954 | 1.39 |
| *HEG1* | 0.005 | 787 | 568 | 1.39 |
| *IL13RA1* | 0.018 | 1866 | 1347 | 1.39 |
| *IRF3* | 0.01 | 286 | 207 | 1.38 |
| *TNFSF12* | 0.01 | 677 | 490 | 1.38 |
| *SMAD4* | 0.001 | 726 | 526 | 1.38 |
| *AKT3* | 0.039 | 876 | 635 | 1.38 |
| *FOXO4* | 0.002 | 323 | 235 | 1.37 |
| *NAA15* | 0.01 | 312 | 227 | 1.37 |
| *CREBBP* | 0.001 | 489 | 356 | 1.37 |
| *DENND5A* | 0.002 | 381 | 278 | 1.37 |
| *NID2* | 0.04 | 541 | 395 | 1.37 |
| *LAMP1* | 0.025 | 5484 | 4006 | 1.37 |
| *APP* | 0.003 | 5250 | 3838 | 1.37 |
| *NRP2* | 0.031 | 893 | 653 | 1.37 |
| *RORB* | 0.035 | 34.5 | 25.4 | 1.36 |
| *TGFBR2* | 0.001 | 1952 | 1439 | 1.36 |
| *EIF2AK3* | 0.002 | 335 | 248 | 1.35 |
| *ILF3* | 0.02 | 1752 | 1298 | 1.35 |
| *RAMP2* | 0.016 | 152 | 113 | 1.35 |
| *PIK3R2* | 0.002 | 504 | 375 | 1.34 |
| *LAMA4* | 0.015 | 1017 | 761 | 1.34 |
| *TNFRSF1A* | 0.029 | 1432 | 1078 | 1.33 |
| *NME4* | 0.011 | 308 | 232 | 1.33 |
| *EGLN2* | 0.001 | 757 | 576 | 1.31 |
| *TFE3* | 0.032 | 138 | 106 | 1.30 |
| *NOTCH1* | 0.007 | 294 | 226 | 1.30 |
| *MAVS* | 0.036 | 781 | 603 | 1.30 |
| *MAP2K2* | 0.009 | 1077 | 835 | 1.29 |
| *PSMD7* | 0.039 | 1535 | 1191 | 1.29 |
| *DST* | 0.019 | 1236 | 968 | 1.28 |
| *ILK* | 0.023 | 596 | 468 | 1.27 |
| *VEGFB* | 0.044 | 988 | 776 | 1.27 |
| *SETD2* | 0.001 | 616 | 488 | 1.26 |
| *QKI* | 0.038 | 1088 | 862 | 1.26 |
| *A2M* | 0.049 | 4872 | 3863 | 1.26 |
| *IRAK4* | 0.048 | 329 | 263 | 1.25 |
| *MAP2K4* | 0.003 | 257 | 209 | 1.23 |
| *GTF3C1* | 0.027 | 754 | 618 | 1.22 |
| *REPS1* | 0.034 | 549 | 451 | 1.22 |
| *HDAC5* | 0.001 | 391 | 323 | 1.21 |
| *PIN1* | 0.021 | 317 | 263 | 1.21 |
| *EP300* | 0.01 | 615 | 530 | 1.16 |
| *ROCK2* | 0.015 | 860 | 752 | 1.14 |
| *RAC1* | 0.039 | 1803 | 2167 | -1.20 |
| *TMPO* | 0.034 | 329 | 404 | -1.23 |
| *GLYR1* | 0.039 | 244 | 301 | -1.23 |
| *CIB1* | 0.03 | 1048 | 1299 | -1.24 |
| *SLC12A6* | 0.018 | 137 | 172 | -1.26 |
| *CHD4* | 0.006 | 611 | 772 | -1.26 |
| *BCL10* | 0.027 | 717 | 924 | -1.29 |
| *PKNOX1* | 0.033 | 64.5 | 83.7 | -1.30 |
| *VAV2* | 0.01 | 144 | 189 | -1.31 |
| *TICAM2* | 0.024 | 121 | 160 | -1.32 |
| *CD2AP* | 0.045 | 463 | 614 | -1.33 |
| *ATG10* | 0.025 | 80.4 | 107 | -1.33 |
| *RB1* | 0.005 | 297 | 398 | -1.34 |
| *PSEN1* | 0.005 | 332 | 450 | -1.36 |
| *BCL2L1* | 0.005 | 1326 | 1810 | -1.37 |
| *ADAM9* | 0.031 | 672 | 918 | -1.37 |
| *IL15RA* | 0.026 | 233 | 320 | -1.37 |
| *IL12RB1* | 0.048 | 82.2 | 113 | -1.37 |
| *RIPK2* | 0.049 | 236 | 325 | -1.38 |
| *IL15* | 0.002 | 86.4 | 119 | -1.38 |
| *CKLF* | 0.023 | 590 | 814 | -1.38 |
| *FLT1* | 0.04 | 150 | 207 | -1.38 |
| *MICB* | 0.045 | 128 | 177 | -1.38 |
| *LGALS3* | 0.036 | 2230 | 3166 | -1.42 |
| *VAMP8* | 0.007 | 265 | 377 | -1.42 |
| *VASH1* | 0.01 | 111 | 158 | -1.42 |
| *GRHL2* | 0.045 | 88.1 | 126 | -1.43 |
| *NLRC5* | 0.03 | 258 | 371 | -1.44 |
| *ALCAM* | 0.005 | 267 | 387 | -1.45 |
| *CLEC4A* | 0.012 | 125 | 183 | -1.46 |
| *SIGIRR* | 0.017 | 185 | 271 | -1.46 |
| *PLXNC1* | 0.016 | 280 | 411 | -1.47 |
| *TBX1* | 0.03 | 21.5 | 31.6 | -1.47 |
| *SLC37A1* | 0.013 | 80.2 | 118 | -1.47 |
| *PRKCZ* | 0.008 | 71.9 | 106 | -1.47 |
| *SELPLG* | 0.024 | 120 | 177 | -1.48 |
| *PFKFB1* | 0.027 | 24 | 35.6 | -1.48 |
| *SPINT1* | 0.048 | 371 | 552 | -1.49 |
| *NUP107* | 0.001 | 178 | 265 | -1.49 |
| *TLR3* | 0.002 | 70.3 | 105 | -1.49 |
| *TNFRSF4* | 0.016 | 42.9 | 64.7 | -1.51 |
| *SORD* | 0.003 | 346 | 522 | -1.51 |
| *KLK3* | 0.021 | 20 | 30.2 | -1.51 |
| *SDC4* | 0.021 | 1679 | 2539 | -1.51 |
| *ITIH4* | 0.03 | 76.5 | 116 | -1.52 |
| *TAL1* | 0.035 | 46.7 | 70.9 | -1.52 |
| *BATF* | 0.02 | 78.3 | 119 | -1.52 |
| *POPDC3* | 0.043 | 21.8 | 33.5 | -1.54 |
| *RELB* | 0.036 | 136 | 209 | -1.54 |
| *DSC2* | 0.043 | 311 | 481 | -1.55 |
| *CCR1* | 0.015 | 73 | 113 | -1.55 |
| *CDS1* | 0.036 | 139 | 216 | -1.55 |
| *ADAMTS12* | 0.035 | 165 | 257 | -1.56 |
| *ANGPT2* | 0.028 | 74.7 | 117 | -1.57 |
| *PRG2* | 0.036 | 25.2 | 39.6 | -1.57 |
| *CD47* | 0.001 | 757 | 1193 | -1.58 |
| *PYCARD* | 0.002 | 115 | 182 | -1.58 |
| *WNT5B* | 0.027 | 44.5 | 70.5 | -1.58 |
| *RACGAP1* | 0.003 | 104 | 165 | -1.59 |
| *CARD11* | 0.008 | 150 | 238 | -1.59 |
| *NOD2* | 0.026 | 73.1 | 116 | -1.59 |
| *CCL8* | 0.031 | 24.8 | 39.4 | -1.59 |
| *RBM47* | 0.008 | 187 | 298 | -1.59 |
| *SH2D3A* | 0.006 | 172 | 275 | -1.60 |
| *ADAM8* | 0.037 | 105 | 168 | -1.60 |
| *CCBE1* | 0.029 | 35.2 | 56.5 | -1.61 |
| *LY86* | 0.01 | 68.3 | 110 | -1.61 |
| *AMH* | 0.01 | 26 | 41.9 | -1.61 |
| *CX3CL1* | 0.026 | 124 | 200 | -1.61 |
| *TNFRSF8* | 0.036 | 33.6 | 54.3 | -1.62 |
| *ARAP2* | 0.001 | 109 | 177 | -1.62 |
| *CAMP* | 0.03 | 21.7 | 35.4 | -1.63 |
| *TNF* | 0.03 | 32.1 | 52.5 | -1.64 |
| *TOM1L1* | 0.012 | 160 | 262 | -1.64 |
| *EPHA2* | 0.017 | 393 | 644 | -1.64 |
| *ATG12* | 0.017 | 26 | 42.7 | -1.64 |
| *CCR6* | 0.008 | 42.3 | 69.6 | -1.65 |
| *CD180* | 0.004 | 61.9 | 102 | -1.65 |
| *TNFRSF18* | 0.017 | 55.9 | 92.4 | -1.65 |
| *LAD1* | 0.02 | 423 | 708 | -1.67 |
| *PLA2G3* | 0.046 | 20 | 33.6 | -1.68 |
| *F2RL1* | 0.038 | 250 | 422 | -1.69 |
| *IFI35* | 0.019 | 109 | 185 | -1.70 |
| *EPHA1* | 0.002 | 63.6 | 108 | -1.70 |
| *PPARG* | 0.007 | 97.6 | 166 | -1.70 |
| *HPSE* | 0.002 | 83.4 | 142 | -1.70 |
| *ITGA3* | 0.041 | 797 | 1358 | -1.70 |
| *CHRNA7* | 0.03 | 62.1 | 106 | -1.71 |
| *HUNK* | 0.006 | 31.4 | 53.7 | -1.71 |
| *TREM2* | 0.004 | 51.5 | 88.4 | -1.72 |
| *STAB2* | 0.013 | 20 | 34.4 | -1.72 |
| *CASP1* | 0.001 | 130 | 224 | -1.72 |
| *KLRC1* | 0.041 | 40.6 | 70 | -1.72 |
| *PTK6* | 0.014 | 132 | 228 | -1.73 |
| *SPOCK3* | 0.005 | 20 | 34.8 | -1.74 |
| *CD207* | 0.023 | 25.8 | 45 | -1.74 |
| *IL23A* | 0.046 | 26.4 | 46.1 | -1.75 |
| *INHBE* | 0.029 | 20 | 35.4 | -1.77 |
| *ICAM4* | 0.029 | 23.9 | 42.5 | -1.78 |
| *CD8B* | 0.047 | 57.3 | 102 | -1.78 |
| *SYT17* | 0.005 | 26.7 | 47.6 | -1.78 |
| *ITGB4* | 0.047 | 1349 | 2415 | -1.79 |
| *TGFB2* | 0.015 | 96.4 | 174 | -1.80 |
| *EVPL* | 0.009 | 164 | 297 | -1.81 |
| *IBSP* | 0.001 | 22.4 | 40.6 | -1.81 |
| *EPN3* | 0.014 | 65.6 | 119 | -1.81 |
| *CD58* | 0.001 | 233 | 423 | -1.82 |
| *LAMP3* | 0.002 | 65.8 | 120 | -1.82 |
| *CAMK2A* | 0.014 | 20 | 36.6 | -1.83 |
| *MISP* | 0.028 | 289 | 529 | -1.83 |
| *TLR5* | 0.001 | 55.1 | 101 | -1.83 |
| *CCR4* | 0.006 | 36.6 | 67.5 | -1.84 |
| *OAS3* | 0.005 | 203 | 375 | -1.85 |
| *TBX21* | 0.024 | 33.6 | 62.2 | -1.85 |
| *NODAL* | 0.022 | 21.8 | 40.9 | -1.88 |
| *CD70* | 0.043 | 30.1 | 56.6 | -1.88 |
| *FOXP3* | 0.006 | 33.8 | 63.7 | -1.88 |
| *ADM2* | 0.006 | 49.1 | 92.6 | -1.89 |
| *TLR7* | 0.009 | 36.1 | 68.1 | -1.89 |
| *IFIH1* | 0.001 | 102 | 193 | -1.89 |
| *TMPRSS4* | 0.024 | 297 | 562 | -1.89 |
| *TPSD1* | 0.018 | 23.5 | 44.5 | -1.89 |
| *CASP10* | 0.026 | 26 | 49.3 | -1.90 |
| *IKBKE* | 0.001 | 126 | 239 | -1.90 |
| *CCRL2* | 0.006 | 77.3 | 147 | -1.90 |
| *ROPN1* | 0.038 | 23.4 | 44.5 | -1.90 |
| *SH2D1B* | 0.027 | 23.6 | 44.9 | -1.90 |
| *SPA17* | 0.001 | 87.2 | 166 | -1.90 |
| *PPL* | 0.017 | 221 | 422 | -1.91 |
| *CLDN1* | 0.024 | 301 | 578 | -1.92 |
| *CDKN2A* | 0.029 | 21.5 | 41.3 | -1.92 |
| *CLEC7A* | 0.001 | 90.5 | 174 | -1.92 |
| *CD86* | 0.002 | 56 | 108 | -1.93 |
| *PITX2* | 0.031 | 23.3 | 45.3 | -1.94 |
| *GDF5* | 0.03 | 21 | 41.1 | -1.96 |
| *AIRE* | 0.028 | 23.4 | 45.9 | -1.96 |
| *CD160* | 0.03 | 23.4 | 45.9 | -1.96 |
| *HKDC1* | 0.025 | 99.3 | 195 | -1.96 |
| *CBLC* | 0.024 | 32.3 | 63.5 | -1.97 |
| *F12* | 0.001 | 32.6 | 64.1 | -1.97 |
| *CCR3* | 0.015 | 23.9 | 47.1 | -1.97 |
| *CD274* | 0.019 | 43.4 | 86.4 | -1.99 |
| *IFIT2* | 0.001 | 107 | 214 | -2.00 |
| *UTS2* | 0.013 | 20 | 40.2 | -2.01 |
| *OAS1* | 0.005 | 331 | 669 | -2.02 |
| *PRAME* | 0.016 | 28.1 | 56.8 | -2.02 |
| *DEFB1* | 0.006 | 29.3 | 59.6 | -2.03 |
| *CHAD* | 0.005 | 23.1 | 47.3 | -2.05 |
| *HOXA7* | 0.017 | 20.1 | 41.2 | -2.05 |
| *MUC1* | 0.045 | 1594 | 3277 | -2.06 |
| *CCL26* | 0.009 | 31.7 | 65.2 | -2.06 |
| *CD80* | 0.001 | 39.3 | 81 | -2.06 |
| *C9* | 0.024 | 23.4 | 48.4 | -2.07 |
| *CTCFL* | 0.035 | 23.4 | 48.7 | -2.08 |
| *LTA* | 0.011 | 26.3 | 54.9 | -2.09 |
| *KIR3DL2* | 0.029 | 23.4 | 49.3 | -2.11 |
| *IL21R* | 0.008 | 34.1 | 72.7 | -2.13 |
| *ICOS* | 0.008 | 31.1 | 66.8 | -2.15 |
| *BIRC5* | 0.002 | 111 | 239 | -2.15 |
| *IRAK2* | 0.006 | 94 | 203 | -2.16 |
| *XCL2* | 0.034 | 32 | 69.2 | -2.16 |
| *IFI27* | 0.014 | 2480 | 5366 | -2.16 |
| *ITGA2* | 0.011 | 264 | 576 | -2.18 |
| *MTBP* | 0.001 | 33.9 | 74.1 | -2.19 |
| *IFNG* | 0.032 | 21.7 | 47.6 | -2.19 |
| *SMOC1* | 0.014 | 27.6 | 60.8 | -2.20 |
| *TDGF1* | 0.015 | 22.2 | 49.4 | -2.23 |
| *IRF6* | 0.023 | 233 | 527 | -2.26 |
| *CXCL6* | 0.027 | 146 | 331 | -2.27 |
| *IL2RG* | 0.009 | 200 | 461 | -2.31 |
| *SLC2A1* | 0.008 | 125 | 289 | -2.31 |
| *IL7* | 0.003 | 29.3 | 68.9 | -2.35 |
| *PTTG1* | 0.001 | 176 | 414 | -2.35 |
| *CEACAM6* | 0.036 | 2857 | 6746 | -2.36 |
| *MET* | 0.001 | 176 | 419 | -2.38 |
| *OCLN* | 0.01 | 65.1 | 157 | -2.41 |
| *PBK* | 0.005 | 37.3 | 91.1 | -2.44 |
| *IL18* | 0.001 | 143 | 351 | -2.45 |
| *S100A7* | 0.024 | 29.1 | 71.9 | -2.47 |
| *CDK1* | 0.003 | 93.8 | 232 | -2.47 |
| *TTK* | 0.001 | 31.8 | 80.6 | -2.53 |
| *KRT7* | 0.009 | 900 | 2316 | -2.57 |
| *KRT19* | 0.01 | 1398 | 3689 | -2.64 |
| *S100A14* | 0.006 | 202 | 552 | -2.73 |
| *IFIT1* | 0.004 | 107 | 294 | -2.75 |
| *RRAD* | 0.034 | 54.9 | 151 | -2.75 |
| *LAMC2* | 0.013 | 450 | 1247 | -2.77 |
| *SPINK5* | 0.02 | 27.1 | 75.7 | -2.79 |
| *KISS1* | 0.004 | 23.8 | 67.2 | -2.82 |
| *CXCL5* | 0.045 | 355 | 1030 | -2.90 |
| *TACSTD2* | 0.002 | 1417 | 4325 | -3.05 |
| *CCL18* | 0.028 | 215 | 661 | -3.07 |
| *LAMB3* | 0.007 | 346 | 1093 | -3.16 |
| *IFNL2* | 0.009 | 23.6 | 75.6 | -3.20 |
| *ITGB6* | 0.044 | 160 | 513 | -3.21 |
| *LAMA3* | 0.006 | 157 | 504 | -3.21 |
| *ULBP2* | 0.004 | 24.4 | 88.7 | -3.64 |
| *MUC16* | 0.024 | 66.5 | 261 | -3.92 |
| *CT45A1* | 0.026 | 23.4 | 116 | -4.96 |
| *IFNA7* | 0.033 | 23.4 | 116 | -4.96 |

**Table S3:** List of genes with Variable Importance in Projection (VIP) score in PLS model of more than 1.0. Area under the receiver operating curve (AUROC) values were determined by univariate logistic regression to determine the diagnostic ability of the gene.

| **Increased Expression** | | | |  | **Decreased Expression** | | | |
| --- | --- | --- | --- | --- | --- | --- | --- | --- |
| **Gene** | **VIP** | **AUROC** | **FC** |  | **Gene** | **VIP** | **AUROC** | **FC** |
| *ABL1* | 1.016 | 0.939 | 2.153 |  | *IL18* | 1.473 | 0.926 | -2.455 |
| *SFRP1* | 1.468 | 0.916 | 3.698 |  | *SPA17* | 1.317 | 0.862 | -1.904 |
| *CHRDL1* | 1.584 | 0.916 | 2.446 |  | *CD58* | 1.198 | 0.844 | -1.815 |
| *IGF1* | 1.505 | 0.908 | 3.067 |  | *PTTG1* | 1.098 | 0.841 | -2.352 |
| *CFD* | 1.496 | 0.890 | 2.599 |  | *MTBP* | 1.213 | 0.835 | -2.186 |
| *IGFBP4* | 1.473 | 0.887 | 2.102 |  | *IFIH1* | 1.071 | 0.834 | -1.892 |
| *OGN* | 1.286 | 0.882 | 3.727 |  | *ARAP2* | 1.231 | 0.829 | -1.624 |
| *CCDC80* | 1.358 | 0.877 | 2.203 |  | *CD47* | 1.459 | 0.826 | -1.576 |
| *MAPK14* | 1.007 | 0.875 | 1.587 |  | *IFIT1* | 1.240 | 0.824 | -2.748 |
| *LTBP4* | 1.334 | 0.872 | 2.164 |  | *IFIT2* | 1.216 | 0.824 | -2.000 |
| *MAPKAPK2* | 1.131 | 0.870 | 1.675 |  | *NUP107* | 1.153 | 0.824 | -1.489 |
| *FHL1* | 1.099 | 0.867 | 2.479 |  | *CASP1* | 1.242 | 0.821 | -1.723 |
| *FGF2* | 1.407 | 0.867 | 2.253 |  | *IKBKE* | 1.048 | 0.811 | -1.897 |
| *FBLN5* | 1.223 | 0.864 | 2.195 |  | *F12* | 1.042 | 0.806 | -1.966 |
| *TNXB* | 1.252 | 0.862 | 2.622 |  | *TTK* | 1.137 | 0.803 | -2.535 |
| *CXCL12* | 1.495 | 0.859 | 2.356 |  | *CD80* | 1.125 | 0.803 | -2.061 |
| *ADAMTS1* | 1.395 | 0.859 | 2.228 |  | *MET* | 1.066 | 0.801 | -2.381 |
| *BAG2* | 1.495 | 0.859 | 1.769 |  | *CLEC7A* | 1.403 | 0.801 | -1.923 |
| *CD81* | 1.204 | 0.859 | 1.586 |  | *CCL18* | 1.069 | 0.798 | -3.074 |
| *GPR124* | 1.169 | 0.857 | 1.842 |  | *BIRC5* | 1.082 | 0.795 | -2.153 |
| *DLC1* | 1.161 | 0.857 | 1.624 |  | *TLR5* | 1.503 | 0.795 | -1.833 |
| *CSPG4* | 1.166 | 0.852 | 2.641 |  | *IBSP* | 1.108 | 0.788 | -1.813 |
| *BCL6* | 1.113 | 0.852 | 1.981 |  | *CD86* | 1.100 | 0.785 | -1.929 |
| *SETD2* | 1.184 | 0.852 | 1.262 |  | *ULBP2* | 1.094 | 0.783 | -3.635 |
| *JAM2* | 1.236 | 0.849 | 1.881 |  | *CDK1* | 1.052 | 0.783 | -2.473 |
| *IL6ST* | 1.002 | 0.847 | 1.751 |  | *OAS3* | 1.174 | 0.783 | -1.847 |
| *MEG3* | 1.261 | 0.844 | 1.856 |  | *HPSE* | 1.010 | 0.777 | -1.703 |
| *MPDZ* | 1.300 | 0.843 | 1.643 |  | *EPHA1* | 1.145 | 0.777 | -1.698 |
| *PTX3* | 1.241 | 0.841 | 5.654 |  | *SORD* | 1.218 | 0.775 | -1.509 |
| *ZCCHC24* | 1.284 | 0.839 | 1.919 |  | *LAMP3* | 1.068 | 0.772 | -1.824 |
| *FSTL1* | 1.244 | 0.839 | 1.648 |  | *DEFB1* | 1.156 | 0.760 | -2.034 |
| *FMOD* | 1.298 | 0.839 | 1.489 |  | *TLR3* | 1.061 | 0.754 | -1.494 |
| *CD99* | 1.052 | 0.836 | 1.786 |  | *TREM2* | 1.003 | 0.752 | -1.717 |
| *COL6A1* | 1.444 | 0.836 | 1.716 |  | *PYCARD* | 1.054 | 0.752 | -1.583 |
| *MFAP4* | 1.166 | 0.834 | 2.081 |  | *BCL2L1* | 1.200 | 0.747 | -1.365 |
| *CAMK2D* | 1.179 | 0.834 | 1.453 |  | *CLEC4A* | 1.203 | 0.744 | -1.464 |
| *ITGA7* | 1.093 | 0.831 | 2.570 |  | *IL15* | 1.161 | 0.744 | -1.377 |
| *SPARCL1* | 1.207 | 0.831 | 1.801 |  | *KRT7* | 1.014 | 0.729 | -2.573 |
| *COL6A2* | 1.318 | 0.831 | 1.739 |  | *PLXNC1* | 1.255 | 0.726 | -1.468 |
| *FBLN1* | 1.133 | 0.829 | 2.091 |  | *AMH* | 1.623 | 0.724 | -1.612 |
| *STAT3* | 1.030 | 0.829 | 1.672 |  | *TGFB2* | 1.519 | 0.719 | -1.805 |
| *CREBBP* | 1.141 | 0.826 | 1.374 |  | *CCBE1* | 1.208 | 0.711 | -1.605 |
| *ABI3BP* | 1.206 | 0.824 | 2.014 |  | *ALCAM* | 1.096 | 0.708 | -1.449 |
| *CRISPLD2* | 1.155 | 0.824 | 1.723 |  | *CKLF* | 1.019 | 0.708 | -1.380 |
| *TNS1* | 1.144 | 0.821 | 1.950 |  | *MUC16* | 1.052 | 0.703 | -3.925 |
| *NRP1* | 1.143 | 0.821 | 1.516 |  | *IL7* | 1.079 | 0.701 | -2.352 |
| *LIFR* | 1.065 | 0.818 | 1.754 |  | *SIGIRR* | 1.232 | 0.701 | -1.465 |
| *TEK* | 1.214 | 0.816 | 1.668 |  | *IL23A* | 1.092 | 0.680 | -1.746 |
| *FOXO4* | 1.015 | 0.816 | 1.374 |  | *RIPK2* | 1.078 | 0.657 | -1.377 |
| *EMILIN1* | 1.172 | 0.813 | 1.897 |  | *S100A7* | 1.054 | 0.639 | -2.471 |
| *LAMC1* | 1.088 | 0.813 | 1.416 |  | *CT45A1* | 1.009 | 0.629 | -4.957 |
| *FERMT2* | 1.110 | 0.811 | 1.610 |  | *XCL2* | 1.105 | 0.552 | -2.163 |
| *SYNE1* | 1.156 | 0.806 | 1.500 |  |  |  |  |  |
| *FOS* | 1.346 | 0.803 | 2.531 |  |  |  |  |  |
| *PTGIS* | 1.127 | 0.803 | 1.820 |  |  |  |  |  |
| *SMAD4* | 1.157 | 0.803 | 1.380 |  |  |  |  |  |
| *SRF* | 1.067 | 0.801 | 1.524 |  |  |  |  |  |
| *PDCL3* | 1.154 | 0.801 | 1.476 |  |  |  |  |  |
| *TGFBR2* | 1.087 | 0.801 | 1.356 |  |  |  |  |  |
| *EGLN2* | 1.135 | 0.801 | 1.314 |  |  |  |  |  |
| *AKAP2* | 1.116 | 0.798 | 1.528 |  |  |  |  |  |
| *CD34* | 1.047 | 0.795 | 1.544 |  |  |  |  |  |
| *PIK3R1* | 1.138 | 0.795 | 1.459 |  |  |  |  |  |
| *ILF3* | 1.036 | 0.793 | 1.350 |  |  |  |  |  |
| *PLA2G6* | 1.149 | 0.788 | 1.646 |  |  |  |  |  |
| *EIF2AK3* | 1.589 | 0.788 | 1.351 |  |  |  |  |  |
| *ADAMTS8* | 1.164 | 0.785 | 3.372 |  |  |  |  |  |
| *ROBO4* | 1.259 | 0.785 | 1.646 |  |  |  |  |  |
| *ID4* | 1.145 | 0.785 | 1.615 |  |  |  |  |  |
| *PECAM1* | 1.140 | 0.785 | 1.523 |  |  |  |  |  |
| *HSPG2* | 1.106 | 0.783 | 1.427 |  |  |  |  |  |
| *APP* | 1.121 | 0.783 | 1.368 |  |  |  |  |  |
| *WWTR1* | 1.191 | 0.780 | 1.557 |  |  |  |  |  |
| *SPHK2* | 1.048 | 0.780 | 1.405 |  |  |  |  |  |
| *TNFSF12* | 1.348 | 0.777 | 1.382 |  |  |  |  |  |
| *ZEB1* | 1.242 | 0.775 | 1.615 |  |  |  |  |  |
| *DENND5A* | 1.037 | 0.775 | 1.371 |  |  |  |  |  |
| *HDAC5* | 1.156 | 0.772 | 1.211 |  |  |  |  |  |
| *THBS4* | 1.050 | 0.770 | 2.699 |  |  |  |  |  |
| *MCAM* | 1.082 | 0.770 | 1.791 |  |  |  |  |  |
| *CMA1* | 1.019 | 0.767 | 1.413 |  |  |  |  |  |
| *PIK3R2* | 1.053 | 0.767 | 1.344 |  |  |  |  |  |
| *RORA* | 1.187 | 0.765 | 1.465 |  |  |  |  |  |
| *STAB1* | 1.136 | 0.762 | 1.913 |  |  |  |  |  |
| *CCL14* | 1.014 | 0.760 | 2.370 |  |  |  |  |  |
| *RAMP1* | 1.035 | 0.760 | 1.697 |  |  |  |  |  |
| *IL13RA1* | 1.090 | 0.760 | 1.385 |  |  |  |  |  |
| *THBS1* | 1.041 | 0.757 | 2.381 |  |  |  |  |  |
| *DPT* | 1.014 | 0.754 | 2.149 |  |  |  |  |  |
| *BNC2* | 1.010 | 0.754 | 1.788 |  |  |  |  |  |
| *EPAS1* | 1.159 | 0.754 | 1.436 |  |  |  |  |  |
| *NOS3* | 1.039 | 0.744 | 1.594 |  |  |  |  |  |
| *NOTCH1* | 1.107 | 0.742 | 1.301 |  |  |  |  |  |
| *JAM3* | 1.031 | 0.734 | 1.521 |  |  |  |  |  |
| *CR2* | 1.519 | 0.729 | 1.951 |  |  |  |  |  |
| *TWIST2* | 1.043 | 0.729 | 1.585 |  |  |  |  |  |
| *CDKN1A* | 1.109 | 0.726 | 1.905 |  |  |  |  |  |
| *NME4* | 1.086 | 0.724 | 1.328 |  |  |  |  |  |
| *FXYD6* | 1.142 | 0.711 | 1.734 |  |  |  |  |  |
| *CALD1* | 1.194 | 0.711 | 1.476 |  |  |  |  |  |
| *NR4A1* | 1.106 | 0.701 | 2.273 |  |  |  |  |  |
| *VEGFB* | 1.308 | 0.698 | 1.273 |  |  |  |  |  |
| *A2M* | 1.205 | 0.698 | 1.261 |  |  |  |  |  |
| *PDPN* | 1.051 | 0.680 | 1.674 |  |  |  |  |  |
| *JUN* | 1.200 | 0.665 | 1.541 |  |  |  |  |  |
| *TNC* | 1.006 | 0.647 | 2.004 |  |  |  |  |  |
| *NR4A3* | 1.063 | 0.604 | 1.935 |  |  |  |  |  |

**Table S4:** Optimal cut-offs for the identified biomarkers based on maximum Youden Index.

| **Gene** | **Cut-off** | **Sensitivity** | **Specificity** |
| --- | --- | --- | --- |
| *ABL1* | >581.74 | 94.12% | 86.96% |
| *SFRP1* | >209.89 | 88.24% | 82.61% |
| *CHRDL1* | >253.84 | 94.12% | 86.96% |
| *IGF1* | >371.54 | 82.35% | 91.30% |
| *CFD* | >258.50 | 94.12% | 82.61% |
| *IL18* | <257.14 | 94.12% | 78.26% |
| *SPA17* | <121.31 | 82.35% | 73.91% |
| *CD58* | <382.03 | 94.12% | 69.57% |
| *PTTG1* | <273.79 | 88.24% | 69.57% |
| *MTBP* | <45.48 | 88.24% | 73.91% |

**Table S5:** Multivariable Cox Proportional Hazard Analysis.

| **Variable** | **Estimate** | **95% CI (profile likelihood)** | ***p* value** |
| --- | --- | --- | --- |
| Sex [F] | 0.7514 | 0.3515 to 1.541 | 0.4453 |
| Age [Old] | 1.035 | 0.4879 to 2.217 | 0.9282 |
| Stage [Late] | 0.6480 | 0.1446 to 2.067 | 0.5091 |
| Grade [High] | 1.338 | 0.6539 to 2.706 | 0.4170 |
| Lymph Positivity [Positive] | 1.317 | 0.6590 to 2.718 | 0.4419 |
| PTTG1 [Increased] | 1.802 | 0.8582 to 3.876 | 0.1225 |

**Table S6:** Potential upstream regulators based on the gene expression profile of good-NAC responders compared to poor-NAC responders.

| **Upstream Regulator** | **Molecule Type** | **Activation *z*-score** | ***p*-value of overlap** | **Target Molecules in Dataset** |
| --- | --- | --- | --- | --- |
| PGR | ligand-dependent nuclear receptor | 4.041 | 7.95E-14 | *ADAMTS1,BCL6,CCL8,CDK1,CDKN1A,COL6A1,COL6A2,EPHA1,F2RL1,FOS,IFI27,IFI35,IFIT1,IFIT2,ITGB4,MUC1,OAS1,OAS3,PPARG,PRAME,STAT3,TNC,TNF,ZEB1* |
| TGFBR2 | kinase | 3.317 | 7.58E-22 | *ADAM8,BATF,BIRC5,CASP1,CDKN1A,CDKN2A,CHRNA7,CLU,COL1A1,CRISPLD2,CXCL6,EGR1,FGF2,IFNG,JUN,KRT19,KRT7,MAPKAPK2,NOS3,NRP1,PDPN,PITX2,PPARG,PTGIS,RRAD,SERPINE1,SLC2A1,SNAI1,STAT3,TACSTD2,TBX21,TGFB2,THBS1,TNF,TNFRSF18,TNFRSF4* |
| IL10RA | transmembrane receptor | 3.308 | 4.7E-10 | *CD34,DCN,DPT,F13A1,FHL1,FOXP3,FSTL1,HPSE,IFNG,IKBKE,IL23A,IL2RG,KLK3,OCLN,OGN,PCOLCE,SLC2A1,STAT3,THBS4,TNF* |
| WNT5A | cytokine | 3.236 | 1.24E-08 | *CD34,CD80,COL1A1,CXCL12,CXCL6,KISS1,LAMA3,LAMB3,PECAM1,SDC4,SNAI1,STAT3,TLR5,TNF,WWTR1* |
| CG | complex | 3.122 | 2.49E-24 | *ADAMTS1,C1R,C1S,C4A/C4B,CCL8,CD34,CDK1,CDKN1A,CLU,CNN1,CRISPLD2,CX3CL1,CXCL12,CYP1B1,DPT,EGR1,F2RL1,FHL1,FOS,FYN,HPSE,IFNG,IGF1,IGFBP4,IL18,IL7,ITGA2,ITGA3,JUN,KISS1,MAPK14,MAPKAPK2,MCAM,NOS3,NR4A1,NRP1,PECAM1,PPARG,PTX3,SDC4,SLC2A1,TGFB2,TNF,TNS1* |
| ERG | transcription regulator | 3.101 | 5.54E-05 | *CLDN1,FHL1,FYN,KLK3,MET,NOS3,PECAM1,SLIT2,SNAI1,THBS1,ZEB1* |
| IL1RN | cytokine | 3.031 | 3.16E-10 | *CDKN1A,CXCL5,IFI27,IFIH1,IFNG,IGF1,IGFBP4,IL23A,LAMP3,LIFR,OAS1,OAS3,SERPINE1,TNF* |
| MRTFA | transcription regulator | 3.011 | 2.92E-15 | *ACTG2,CAMP,CDKN2A,CNN1,COL3A1,CXCL12,CXCL6,EGR1,FHL1,FOS,MYH11,MYLK,PRG2,RAMP1,SERPINE1,SLIT2,SPOCK3,SRF,TAL1,TGFB2,THBS1,TNC* |
| TGFB3 | growth factor | 2.988 | 2.35E-14 | *ACTG2,CDKN1A,CDKN2A,COL1A1,COL3A1,F2RL1,FGF2,FOS,JUN,MGP,OCLN,SERPINE1,SNAI1,THBS1,TNC,TNS1,ZEB2* |
| miR-155-5p (miRNAs w/seed UAAUGCU) | mature microRNA | 2.935 | 3.93E-07 | *CASP1,CCL18,CD47,CLDN1,IFNG,IKBKE,IL23A,MET,PPL,SERPINE1,TACSTD2,TNF* |
| TREX1 | enzyme | 2.923 | 2.04E-08 | *CASP1,CD274,CD47,CD86,CDKN1A,IFI27,IFIH1,IFIT1,IFIT2,IKBKE,OAS1,OAS3,TNF* |
| SP110 | transcription regulator | 2.828 | 0.000202 | *CLU,IFI27,IFIH1,IFIT1,IL18,OAS1,OAS3,PPARG* |
| MRTFB | transcription regulator | 2.801 | 8.29E-12 | *ACTG2,ADAMTS1,CAMP,CNN1,COL3A1,CXCL12,CXCL6,EGR1,FHL1,MYH11,PRG2,RAMP1,SERPINE1,SLIT2,SRF,TAL1,TGFB2,THBS1* |
| CDKN1A | kinase | 2.79 | 0.000063 | *BIRC5,CD274,CDK1,CDKN1A,CDKN2A,IFNG,PBK,RACGAP1,SERPINE1,TNF,TNFRSF18,TTK* |
| GDF2 | growth factor | 2.781 | 1.56E-05 | *COL1A1,CXCL12,FOS,IGF1,NRP1,RAMP1,SERPINF1,SPARCL1* |
| TP53 | transcription regulator | 2.665 | 4.15E-26 | *ADAM8,ADGRB3,AIRE,AKAP12,ARAP2,ATG12,BIRC5,C9,CARD11,CASP1,CCDC80,CCR4,CD47,CD70,CD81,CDK1,CDKN1A,CDKN2A,CLU,CNN1,COL1A1,COL3A1,COL6A2,CX3CL1,CXCL12,DLC1,EGR1,EMILIN1,EPHA2,FERMT2,FGF2,FHL1,FOS,FOXP3,FSTL1,FYN,IFI35,IFNG,IGF1,IGFBP4,IL21R,IL7,ITGA2,ITGB4,ITGB6,JUN,KLK3,LAMC2,LAMP3,MCAM,MET,MPDZ,MTA1,NOS3,NR4A3,NRP1,OAS1,PBK,PECAM1,PPARG,PTTG1,PYCARD,RACGAP1,RRAD,SERPINE1,SFRP1,SLC2A1,SNAI1,SRF,STAT3,TGFB2,THBS1,THBS4,THY1,TLR7,TNC,TNF,TNFRSF18,TTK,WNT5B,ZEB1,ZEB2* |
| HNF4A | transcription regulator | 2.64 | 0.00545 | *BCL6,C1S,C4A/C4B,CCL14,CD80,CDK1,CDKN1A,CLDN1,CYP1B1,DSC2,EGR1,EPHA2,EVPL,F12,FHL1,FXYD6,HUNK,IGF1,IL6ST,INHBE,IRF6,ITGA2,ITIH4,JUN,KRT7,LAD1,LAMB3,LAMC2,NOD2,OAS3,OCLN,PPARG,PPL,RPS6,SERPINE1,SLC2A1,THY1,TNC* |
| HIVEP1 | transcription regulator | 2.611 | 2.58E-06 | *CARD11,IL18,IRAK2,LTA,RELB,TLR5,TNF* |
| CITED2 | transcription regulator | 2.588 | 2.83E-07 | *CD274,CD80,CD86,CDKN2A,FOXP3,IFI35,IFIH1,IFIT2,NODAL,OAS1,OAS3,PITX2,TLR7,TNF* |
| PTK2 | kinase | 2.58 | 6.37E-07 | *CDKN1A,COL1A1,CSPG4,FOS,OCLN,SNAI1,THBS1,TNC* |
| ROR1 | kinase | 2.58 | 5.33E-08 | *FSTL1,KRT19,SERPINE1,SNAI1,THBS1,TNS1,ZEB1* |
| CBX5 | transcription regulator | 2.53 | 2.44E-08 | *BIRC5,CDKN1A,CXCL5,CYP1B1,IFIT2,ITGA7,KLK3,LAMB3,OAS1,PPARG,PTK6,TACSTD2* |
| MAPK1 | kinase | 2.522 | 8.65E-16 | *AKAP12,BIRC5,C1S,CCDC80,CDKN1A,DCN,EGR1,FOS,FOXP3,IFI27,IFI35,IFIH1,IFIT1,IFIT2,IFNG,JUN,LAD1,LAMP3,NOS3,NR4A1,NRP1,OAS1,OAS3,PITX2,SERPINE1,SNAI1,SRF,TBX21,TNF* |
| TWIST1 | transcription regulator | 2.477 | 4.71E-17 | *ADAMTS1,CDKN1A,CDKN2A,CHAD,CLU,COL1A1,COL6A1,CXCL12,DCN,DPYSL3,EMILIN1,FOS,IGFBP4,MET,PCOLCE,RELB,SNAI1,TBX21,TGFB2,TNF,ZEB1,ZEB2* |
| SMAD3 | transcription regulator | 2.443 | 7.86E-24 | *ADAMTS1,CDKN1A,CDKN2A,CHRDL1,COL1A1,COL3A1,COL6A1,COL6A2,CRISPLD2,EGR1,F12,FOS,FOXP3,IBSP,IFNG,IL23A,JAM2,JUN,KLK3,NODAL,PCOLCE,PTX3,RPS6,SERPINE1,SNAI1,STAT3,TBX21,TDGF1,TGFB2,THBS1,THBS4,TNC,TNF,TPM2,ZEB2* |
| CCN2 | growth factor | 2.429 | 4.91E-07 | *ADAMTS1,BIRC5,COL1A1,DCN,EGR1,EMILIN1,IGF1,JUN,SDC4,SERPINE1* |
| HDL | complex | 2.425 | 3.37E-05 | *CD80,CD86,CX3CL1,NOS3,PTGIS,TNF* |
| CTF1 | cytokine | 2.425 | 1.89E-07 | *FOS,IL6ST,MYH11,MYLK,PPARG,SRF,TNF* |
| USP8 | peptidase | 2.423 | 0.000012 | *CASP1,CD274,IFIT1,IFIT2,MET,OAS1,OAS3* |
| AGTR1 | G-protein coupled receptor | 2.412 | 1.34E-05 | *COL3A1,FOS,JUN,NOS3,SERPINE1,TNF* |
| miR-146a-5p (and other miRNAs w/seed GAGAACU) | mature microRNA | 2.395 | 0.000912 | *CAMP,CCL8,CCR3,IRAK2,TBX21,TNF* |
| STAT6 | transcription regulator | 2.386 | 9.22E-13 | *BAG2,BCL6,CCL26,CCL8,CD80,CDK1,CDKN1A,COL1A1,COL3A1,CR2,EGR1,EPHA2,FOXP3,ICOS,IFIH1,IFNG,IGF1,IL6ST,LIFR,LTA,OAS3,PRG2,SERPINE1,SERPINF1,TBX21,THY1,TNF,TNFRSF8* |
| TWIST2 | transcription regulator | 2.377 | 1.72E-05 | *CDKN1A,FBLN5,SNAI1,TNF,ZEB1,ZEB2* |
| SMAD4 | transcription regulator | 2.307 | 2.02E-11 | *ANGPT2,CCL18,CDKN1A,CDKN2A,CXCL6,FOS,FOXP3,IFNG,IL18,JAM2,MET,PTX3,RGCC,SERPINE1,SNAI1,TBX21,TGFB2,THBS1,TNC,TNF,TPM2* |
| ZEB1 | transcription regulator | 2.289 | 9.5E-11 | *CDKN2A,COL1A1,COL3A1,ITGB4,KRT19,LAMC2,MUC1,NRP1,OAS1,RBM47,SERPINE1,THBS1,ZEB1* |
| ADORA2A | G-protein coupled receptor | 2.264 | 2.75E-05 | *CD81,COL3A1,COL6A1,FOS,IFNG,NR4A1,NR4A3,OCLN,TNF* |
| SLC22A5 | transporter | 2.236 | 2.25E-05 | *CDKN1A,COL3A1,IFNG,LTBP4,TNF* |
| HYAL1 | enzyme | 2.236 | 0.000284 | *CASP1,CDK1,IGFBP4,ITGA7,RAMP1* |
| ACKR2 | G-protein coupled receptor | 2.236 | 0.000214 | *IFIT2,IFNG,OAS1,OAS3,TNF* |
| MAFB | transcription regulator | 2.236 | 0.0113 | *CXCL12,F13A1,FBLN5,IGF1,STAB1* |
| NRG1 | growth factor | 2.231 | 7.9E-14 | *ANGPT2,CDKN1A,COL1A1,CXCL6,EGR1,EPHA2,FGF2,FOS,FSTL1,GDF5,JUN,KLK3,LIFR,MTA1,NR4A1,NR4A3,SERPINE1,SLC2A1,SLIT2,SNAI1,TGFB2,THBS1,TNS1* |
| MYOD1 | transcription regulator | 2.225 | 5.99E-06 | *BCL6,CDK1,CDKN1A,FSTL1,FYN,IFIT2,IGF1,ITGA7,PBK,RACGAP1,SLC2A1,TNS1,ZEB1* |
| MAP2K1 | kinase | 2.187 | 6.77E-09 | *CD274,CDKN1A,CDKN2A,COL1A1,COL3A1,ELK1,FGF2,FOS,ITGB4,JUN,PECAM1,THBS1,TNC,TNF,TREM2* |
| IRGM | enzyme | 2.186 | 0.000284 | *IFI35,IFIH1,IFIT1,IFIT2,OAS1* |
| EPHB1 | kinase | 2.177 | 7.33E-08 | *EGR1,FOS,IL6ST,JUN,SERPINE1* |
| NGLY1 | enzyme | 2.159 | 4.76E-05 | *IFI27,IFIT1,IFIT2,OAS1,OAS3* |
| EPHA2 | kinase | 2.158 | 2.74E-08 | *CD274,EGR1,FGF2,FOS,IL18,JUN,NR4A1,SLIT2* |
| FGFR1 | kinase | 2.154 | 1.71E-08 | *BIRC5,CDKN1A,COL1A1,COL3A1,CRISPLD2,FGF2,FOS,ITGB4,JUN,MFAP4,RPS6,TNC* |
| IGF1R | transmembrane receptor | 2.124 | 2.51E-10 | *CDK1,CDKN1A,CLU,COL1A1,COL3A1,EGR1,FOS,FOXP3,IBSP,IGF1,IGFBP4,KRT19,MAPK14,NOS3,NR4A1,RACGAP1,SDC4,SNAI1,STAT3,TNF* |
| GATA6 | transcription regulator | 2.121 | 2.93E-10 | *AMH,C1R,C4A/C4B,CDKN1A,EVPL,FGF2,IRF6,KRT7,MYH11,MYLK,NODAL,NOS3,PECAM1,PPARG,PPL,PTGIS,TGFB2* |
| ELK1 | transcription regulator | 2.1 | 2.25E-16 | *CDKN1A,CDKN2A,EGR1,ELK1,FOS,IL23A,IL7,ITGB6,JAM2,JUN,MYLK,NR4A1,SLC2A1,SNAI1,THBS1,TNF* |
| MAP3K1 | kinase | 2.095 | 1.46E-07 | *COL3A1,EGR1,FOS,JUN,SERPINE1,THBS1,TNC,TNF* |
| FGF1 | growth factor | 2.04 | 2.48E-07 | *ANGPT2,CDKN1A,CR2,EGR1,FOS,JUN,NOS3,PECAM1,TGFB2,THBS1,TNF* |
| NR3C2 | ligand-dependent nuclear receptor | 2.025 | 1.17E-08 | *ADAMTS1,COL1A1,COL3A1,CXCL6,CYP1B1,EGR1,FGF2,IGF1,NOS3,PTX3,RRAD,SERPINE1,TNF* |
| JAG1 | growth factor | 2.013 | 6.2E-10 | *CD86,CDKN1A,CLDN1,COL1A1,IFNG,LAMC2,LIFR,MYH11,SNAI1,TGFB2,TNC* |
| FBXO44 | enzyme | 2 | 0.00455 | *CD274,IFIH1,SPA17,ULBP2* |
| RNF31 | enzyme | 2 | 0.00144 | *CXCL6,IGFBP4,IL18,IL2RG,TNF* |
| MESP2 | transcription regulator | 2 | 3.23E-05 | *SNAI1,TWIST2,ZEB1,ZEB2* |
| SOX7 | transcription regulator | 2 | 0.00673 | *EVPL,IRF6,KRT7,PPL* |
| EFNA5 | kinase | 2 | 0.00252 | *ITGB4,ITGB6,KRT7,S100A7* |
| NODAL | growth factor | 2 | 0.000252 | *CD34,CD80,CD86,TNF* |
| EFNA4 | kinase | 2 | 0.00122 | *ITGB4,ITGB6,KRT7,S100A7* |
| EFNA3 | kinase | 2 | 0.00133 | *ITGB4,ITGB6,KRT7,S100A7* |
| HBB | transporter | 2 | 2.45E-13 | *BCL6,CXCL5,CXCL6,IFIT2,IFNG,IL7,LTA,MAPK14,MAPKAPK2,PECAM1,RELB,SERPINE1,TGFB2,TLR5,TNF* |
| ACTN4 | transcription regulator | 2 | 6.41E-05 | *DCN,FOS,SERPINE1,SNAI1* |
| SETD2 | enzyme | -2 | 0.00158 | *IGF1,JUN,SERPINE1,SERPINF1* |
| SENP3 | peptidase | -2 | 0.00172 | *CD80,CD86,CD8B,IFIT2* |
| IL18 | cytokine | -2.012 | 4.38E-07 | *BCL6,CAMP,CCR4,CCRL2,CD86,CHRNA7,CXCL12,IFNG,IL18,JUN,TBX21,TNF,ULBP2* |
| IFNAR1 | transmembrane receptor | -2.03 | 3.94E-09 | *CD274,CD86,CXCL6,IFIH1,IFIT2,IFNG,IL18,LAMP3,OAS1,OAS3,SERPINE1,THBS1,TLR7,TNF* |
| CD28 | transmembrane receptor | -2.051 | 2.72E-12 | *CAMK2A,CCR4,CCR6,CD274,CD80,CD86,FOS,FOXP3,FYN,ICOS,IFNG,IL21R,IL23A,IL7,JUN,LTA,NOS3,NR4A1,RGCC,STAT3,TBX21,THBS1,TNF,TNFRSF18* |
| NFkB (complex) | complex | -2.08 | 1.46E-24 | *AMH,BAG2,BATF,BIRC5,C1R,CAMP,CASP1,CCL8,CD274,CD80,CD86,CD99,CDKN1A,CLU,CX3CL1,CXCL12,CXCL5,CXCL6,EGR1,ELK1,F2RL1,FGF2,FOS,FOXP3,ICOS,IFNA7,IFNG,IKBKE,IL18,IL23A,JUN,KRT19,LTA,MTA1,MYLK,PECAM1,PPARG,PTX3,RELB,SDC4,SERPINE1,SLC2A1,SLIT2,SNAI1,TNF,TNFRSF4,TNFSF14* |
| AHR | ligand-dependent nuclear receptor | -2.081 | 1.77E-23 | *CARD11,CCR6,CD274,CD8B,CDK1,CDKN1A,CDKN2A,COL1A1,COL3A1,COL6A1,CYP1B1,DCN,EMILIN1,FBLN5,FOS,FOXP3,HUNK,ICOS,IFNG,IL21R,ITGA7,ITGB6,ITIH4,JUN,LAMA3,LAMC2,MCAM,MGP,MYH11,OCLN,PPARG,PTX3,SERPINE1,SLC2A1,SNAI1,STAT3,TGFB2,THBS1,TLR5,TNF,TNFRSF18* |
| IL33 | cytokine | -2.083 | 3.08E-19 | *ADAMTS1,BATF,CASP1,CCL18,CCR1,CCR4,CD70,CD80,CD86,CLEC7A,COL3A1,CX3CL1,FOXP3,ICOS,IFNG,IKBKE,IL2RG,ITGB4,MYLK,NR4A1,NRP1,OCLN,PPARG,PRG2,RAMP1,RELB,SDC4,SERPINE1,TNF,TNFRSF18,TNFRSF4,TNFSF14,TPSD1,ZEB2* |
| SMAD7 | transcription regulator | -2.109 | 2.42E-10 | *CDK1,CDKN1A,COL1A1,COL3A1,COL6A1,CXCL12,DCN,IFNG,ITGA7,MET,PITX2,SERPINE1,TGFB2,TNF,TPM2* |
| DPP4 | peptidase | -2.114 | 1.18E-05 | *CD86,FGF2,IFNG,LRP1,THBS1,TNF* |
| REL | transcription regulator | -2.125 | 6.15E-13 | *BIRC5,CD80,CD86,CDKN1A,CR2,FOS,FOXP3,IFNG,IL18,IL23A,IRF6,JUN,NR4A1,NR4A3,PYCARD,RELB,SNAI1,STAT3,TNF,TNFRSF4,TTK* |
| TLR2 | transmembrane receptor | -2.131 | 8.84E-10 | *CCR1,CD70,CD80,CD86,CDKN1A,CDKN2A,CXCL6,FOS,FOXP3,IFNG,IFNL2,IL18,IL23A,PTX3,TBX21,TNF* |
| PRKD1 | kinase | -2.131 | 1.41E-06 | *BIRC5,CD86,CXCL6,IBSP,IFNG,IL23A,NR4A1,TNF* |
| CYLD | transcription regulator | -2.147 | 7.67E-10 | *CD80,CD86,CR2,ICOS,IFNG,JUN,RELB,RPS6,SERPINE1* |
| ZBTB10 | transcription regulator | -2.154 | 8.76E-17 | *ADAM8,BIRC5,CD207,CD274,CD34,CD80,CD86,CX3CL1,CXCL5,HPSE,IFIH1,IFIT1,IFIT2,IL23A,LRP1,LTA,NR4A3,OAS1,SERPINE1,TNF,TNFRSF8* |
| TNFSF15 | cytokine | -2.174 | 8.01E-05 | *CD80,CD86,IFNG,TBX21,TNF* |
| RNASE2 | enzyme | -2.179 | 5.45E-05 | *CCL8,CD86,CXCL5,IL7,TNF* |
| BTK | kinase | -2.182 | 5.26E-12 | *CD274,CD86,CDKN1A,CDKN2A,ICOS,IFI35,IFIT1,IFNG,IFNL2,IL18,IL21R,JUN,OAS3,RELB,TNF* |
| ITGA6 | transmembrane receptor | -2.19 | 1.71E-06 | *BIRC5,ITGA2,ITGA3,ITGA7,ITGB4* |
| TYROBP | transmembrane receptor | -2.194 | 4.39E-07 | *CD86,IFNG,IL18,ITGB4,NOD2,RBM47,TNF,TREM2* |
| TRAF6 | enzyme | -2.197 | 0.00153 | *CD274,CD80,CXCL5,RELB,TNF* |
| TLR3 | transmembrane receptor | -2.207 | 4.21E-14 | *CD274,CD70,CD80,CD86,CLU,CX3CL1,DEFB1,FOS,IFIH1,IFIT1,IFIT2,IFNA7,IFNG,IFNL2,IL23A,NR4A1,OAS1,PECAM1,PTX3,SERPINE1,TLR7,TNC,TNF* |
| IL23 | complex | -2.208 | 1.72E-05 | *CCR6,ICOS,IFNG,STAT3,TBX21,TNF* |
| IL5 | cytokine | -2.212 | 1.94E-07 | *BCL6,CCR1,CCR3,CCRL2,CD80,CDKN1A,EGR1,IFNG,IL2RG,ITGB6,LTA,SLC2A1,THY1,TNF,TNFRSF4,TNFRSF8* |
| ORAI1 | ion channel | -2.213 | 2.27E-06 | *CDKN1A,IFNG,PLA2G6,TBX21,TNF* |
| ADAMTS18 | peptidase | -2.213 | 0.000019 | *ITGA3,ITGB4,LAMA3,LAMB3,LAMC2* |
| EIF3E | translation regulator | -2.213 | 0.000127 | *FGF2,SNAI1,THBS1,TNC,ZEB2* |
| PTPRJ | phosphatase | -2.219 | 4.57E-05 | *CD70,FOS,IL18,LTA,MET,TNF* |
| IFNK | cytokine | -2.228 | 4.78E-06 | *CD80,CD86,IFIH1,IFNG,OAS1* |
| MRGPRX3 | G-protein coupled receptor | -2.236 | 0.000142 | *CCR1,CCRL2,CD274,CLEC7A,TNF* |
| PARP2 | enzyme | -2.236 | 9.1E-06 | *CCR6,CD80,CD86,PPARG,TBX21* |
| IKBKE | kinase | -2.279 | 6.68E-09 | *CXCL12,CXCL6,IFIH1,IFIT1,IFIT2,IFNL2,IKBKE,IL23A,IL7,SLC2A1,TNF* |
| IL12 (complex) | complex | -2.292 | 2.82E-12 | *CASP1,CCR4,CCR6,CD86,CXCL6,FGF2,FOS,FOXP3,ICOS,IFIH1,IFIT2,IFNG,IL18,JUN,KLRC1,LTA,RRAD,TBX21,TNF* |
| IL7 | cytokine | -2.335 | 1.13E-07 | *CD274,CD86,CD8B,CDKN1A,COL1A1,FOXP3,ICOS,IFNG,IL7,LTA,SLC2A1,TNF* |
| PTGER2 | G-protein coupled receptor | -2.356 | 4.91E-07 | *EGR1,FOXP3,IFNG,IL23A,PBK,RACGAP1,SERPINE1,THBS1,TNF,TTK* |
| USP22 | peptidase | -2.393 | 5.24E-06 | *CD80,CDKN1A,CFD,FOXP3,IFIT1,TBX21,TNF* |
| DACH1 | transcription regulator | -2.415 | 3.88E-07 | *CDKN1A,EGR1,FGF2,FOS,JUN,KLK3,SERPINE1* |
| INHA | growth factor | -2.433 | 1.43E-11 | *ACTG2,ADAMTS1,CNN1,COL3A1,COL6A1,CYP1B1,IFNG,IGFBP4,ITGA3,JAM2,KRT19,MYH11,PTX3,SERPINE1* |
| CD3 | complex | -2.503 | 2.42E-21 | *ACTG2,BATF,BCL6,BIRC5,C1R,CAMK2A,CCL18,CCR1,CCR4,CCR6,CD274,CD80,CD86,CD8B,CDK1,FOS,FOXP3,FYN,ICOS,ID4,IFI35,IFIT1,IFNG,IGF1,IL21R,IL2RG,JUN,LRP1,LTA,MTA1,NOS3,NR4A1,OGN,PPARG,RELB,TBX21,THBS1,THY1,TNF,TNFRSF18,TNFRSF4,TNFRSF8,TOM1L1* |
| BCL11B | transcription regulator | -2.542 | 1.27E-06 | *BAG2,CDKN1A,FOXP3,IFNG,LTA,NRP1,TBX21,TNF* |
| ITPR2 | ion channel | -2.543 | 9.83E-09 | *ADAM8,CD180,CD274,CDKN2A,CLEC7A,CXCL6,STAB1,TLR5,TLR7,TNF* |
| EBI3 | cytokine | -2.556 | 3.71E-06 | *CCR6,CD274,CD80,CD86,FOS,IFNG,TBX21* |
| KRAS | enzyme | -2.603 | 4.98E-30 | *BIRC5,CASP1,CCDC80,CD274,CD80,CDK1,CDKN1A,CDKN2A,CLU,COL1A1,COL3A1,CX3CL1,CXCL6,DLC1,EGR1,EPHA2,F2RL1,FOS,FOXP3,FSTL1,IFIT1,IFNG,ITGB6,JUN,LAMA3,LAMB3,LAMC2,LAMP3,LTBP4,MAPK14,MCAM,MET,MUC16,MYLK,NOS3,NR4A1,OAS1,OCLN,PCOLCE,PDPN,PECAM1,PTTG1,PYCARD,RPS6,S100A14,SDC4,SERPINE1,SLC2A1,SNAI1,STAT3,TACSTD2,TBX21,THBS1,THY1,TNC,TNF,TTK,WWTR1,ZEB1* |
| IFNA2 | cytokine | -2.674 | 9.21E-16 | *BIRC5,C1R,C1S,CCL8,CD274,CD70,CD86,CDKN1A,CDKN2A,FGF2,IFI27,IFI35,IFIH1,IFIT1,IFIT2,IFNG,LAMP3,MET,NOS3,OAS1,OAS3,PTTG1,TBX21,TNF* |
| TLR7 | transmembrane receptor | -2.736 | 5.36E-11 | *CD274,CD70,CD80,CD86,CDKN1A,FGF2,IFI35,IFIT1,IFNA7,IFNG,IFNL2,IL21R,IL23A,LTA,OAS3,PTX3,TLR7,TNF* |
| IRF5 | transcription regulator | -2.737 | 6.28E-07 | *CDKN1A,COL1A1,COL3A1,IFIH1,IFIT1,IFIT2,IL23A,OAS1,TNF* |
| IFNA4 | cytokine | -2.764 | 9.19E-08 | *CCL8,CCRL2,CD274,CD86,IFIH1,IFIT1,IFIT2,IFNG* |
| CSF2 | cytokine | -2.923 | 2.24E-23 | *ADAM8,BIRC5,C4A/C4B,CARD11,CASP1,CCL18,CCR1,CCRL2,CD180,CD207,CD80,CD86,CDK1,CDKN1A,CLEC7A,EGR1,F2RL1,FOS,FOXP3,ICOS,IFNG,IGF1,IL23A,JUN,LAMP3,LTA,MET,NR4A1,NR4A3,NRP1,PECAM1,PPARG,RACGAP1,RELB,RGCC,SLC2A1,THBS1,TLR5,TNF,TNFRSF18,TNFRSF4,TNFSF14* |
| PRKAA2 | kinase | -2.941 | 4.12E-06 | *CDKN1A,CXCL12,EGR1,IGFBP4,MYLK,PCOLCE,PPARG,SNAI1,TNF,TNS1* |
| FLT3LG | cytokine | -2.947 | 9.83E-09 | *BIRC5,CD274,CD80,CD86,CXCL12,FOXP3,IFNG,TAL1,TNF,TNFRSF18* |
| IL2 | cytokine | -2.964 | 5.02E-23 | *ADAM8,BATF,BCL6,CASP1,CCR1,CCR3,CCR4,CCR6,CD160,CD274,CD47,CD58,CD80,CD86,CD8B,CDK1,CDKN1A,CXCL12,FGF2,FOS,FOXP3,FYN,ICOS,IFNG,IGFBP4,IL18,IL2RG,ITGA2,JUN,KLRC1,LIFR,LTA,MAPKAPK2,NR4A3,NRP1,PECAM1,PPARG,RGCC,RPS6,SERPINE1,SORD,STAT3,TBX21,TNF,TNFRSF18,TNFRSF4,TNFRSF8,TNFSF14,XCL2* |
| IL27 | cytokine | -2.99 | 1.23E-11 | *CCR6,CD274,CD80,CD86,CX3CL1,EGR1,FOS,FOXP3,ICOS,IFNG,IL18,OAS1,TBX21,TNF,TNFRSF4,TNFSF14* |
| FAS | transmembrane receptor | -2.993 | 2.87E-15 | *ARAP2,BIRC5,CCR1,CCRL2,CD80,CD86,CDKN1A,COL1A1,COL3A1,COL6A1,CXCL6,EGR1,F2RL1,FOS,IFIT1,IFNG,IL18,ITGA7,JUN,LAMP3,MET,MGP,NR4A1,PECAM1,PTX3,SERPINE1,TNC,TNF* |
| BHLHE40 | transcription regulator | -3.011 | 2.01E-11 | *BIRC5,CASP1,CCR1,CCRL2,CD274,CD47,CD80,CD86,IFIH1,IFNG,IL2RG,IL6ST,IRAK2,MET,PDPN,SDC4,SLC2A1,SPARCL1,STAT3,THY1,TNF,TNFSF14* |
| STAT1 | transcription regulator | -3.066 | 2.51E-28 | *ANGPT2,BCL6,BIRC5,C1R,C1S,C4A/C4B,CASP1,CCR1,CCR6,CCRL2,CD274,CD86,CDKN1A,COL1A1,COL3A1,CX3CL1,EGR1,FGF2,FOS,FOXP3,IFI27,IFI35,IFIH1,IFIT1,IFIT2,IFNG,IFNL2,IGF1,IL18,IL23A,ITGA2,JUN,MUC1,OAS1,OAS3,PECAM1,PPARG,STAT3,TBX21,TGFB2,THBS4,TNC,TNF* |
| IFNA1/IFNA13 | cytokine | -3.078 | 8.36E-10 | *CCL8,CD274,CD80,CD86,IFI27,IFIH1,IFIT1,IFIT2,IFNG,OAS1* |
| IFNL1 | cytokine | -3.101 | 4.82E-08 | *CD80,IFI27,IFI35,IFIH1,IFIT1,IFIT2,IFNL2,LAMP3,OAS1,OAS3* |
| NONO | transcription regulator | -3.132 | 3.13E-07 | *CCL8,CD80,CDKN2A,IFI27,IFIH1,IFIT1,IFIT2,IKBKE,NOS3,OAS1,OAS3* |
| IRF1 | transcription regulator | -3.139 | 2.82E-13 | *ADAM8,C1R,CASP1,CD274,CD80,CDKN1A,FOXP3,IFI27,IFI35,IFIH1,IFIT1,IFIT2,IFNG,IL18,IL23A,IL7,OAS1,OAS3,STAT3,TNF* |
| FKBP10 | enzyme | -3.162 | 1.4E-09 | *COL1A1,COL3A1,COL6A1,COL6A2,CRISPLD2,IGF1,PDPN,SERPINE1,SNAI1,THY1* |
| IL15 | cytokine | -3.169 | 1.69E-14 | *ACTG2,CCL18,CCR1,CD160,CD274,CD80,CD86,CD8B,CDKN1A,CHRNA7,CXCL12,FOXP3,FYN,ICOS,ID4,IFI35,IFIT1,IFNG,IL21R,IL2RG,JUN,KLRC1,LTA,OGN,RPS6,SERPINE1,TBX21,TNF,TNFRSF18,ULBP2* |
| TNF | cytokine | -3.323 | 6.55E-65 | *ADAM8,ADAMTS8,AKAP12,AMH,ANGPT2,BCL6,BIRC5,C4A/C4B,CAMP,CASP1,CASP10,CCDC80,CCL18,CCL26,CCR1,CCR3,CCR4,CCR6,CCRL2,CD207,CD274,CD34,CD47,CD70,CD80,CD86,CDKN1A,CDKN2A,CFD,CHRNA7,CLDN1,CLU,CNN1,COL1A1,COL3A1,CRISPLD2,CX3CL1,CXCL12,CXCL5,CXCL6,CYP1B1,DCN,DSC2,EGR1,ELK1,EPHA2,F2RL1,FGF2,FOS,FOXP3,FYN,ICOS,IFI27,IFIH1,IFIT1,IFIT2,IFNG,IFNL2,IGF1,IGFBP4,IKBKE,IL18,IL21R,IL23A,IL6ST,IL7,IRAK2,ITGA2,ITGB6,JUN,KLK3,LAD1,LAMA3,LAMB3,LAMC2,LAMP3,LIFR,LTA,MAPK14,MCAM,MET,MGP,MUC1,MYLK,NOD2,NOS3,NR4A1,NR4A3,NRP1,OAS1,OAS3,OCLN,OGN,PDPN,PECAM1,PLA2G3,PPARG,PTGIS,PTX3,PYCARD,RAMP1,RELB,RGCC,RRAD,S100A7,SDC4,SERPINE1,SERPINF1,SFRP1,SLC2A1,SNAI1,STAT3,TBX21,TEK,TGFB2,THBS1,THY1,TLR5,TLR7,TNC,TNF,TNFRSF18,TNFRSF4,TNFRSF8,TNFSF14,TREM2,TWIST2,WWTR1* |
| IFNB1 | cytokine | -3.371 | 8.06E-16 | *AIRE,AMH,CASP1,CD274,CD80,CD86,CDKN1A,CX3CL1,CXCL6,DEFB1,FGF2,FOS,IFI27,IFIH1,IFIT1,IFIT2,IFNG,IL18,NOD2,OAS1,OGN,SH2D1B,THBS1,TLR7,TNF,TNFRSF4* |
| IRF7 | transcription regulator | -3.375 | 6.92E-07 | *CCL8,CCRL2,CD80,FOXP3,IFI35,IFIH1,IFIT1,IFIT2,IFNA7,IFNL2,OAS1,OAS3* |
| TLR4 | transmembrane receptor | -3.466 | 6.99E-16 | *BATF,CCL8,CCRL2,CD274,CD70,CD80,CD86,CXCL6,FOS,HPSE,IFIT2,IFNA7,IFNG,IL18,IL23A,LTA,MET,NOD2,NOS3,NR4A1,NR4A3,PDPN,PPARG,PTX3,RELB,RGCC,SLC2A1,STAT3,TNF,TREM2* |
| TLR9 | transmembrane receptor | -3.591 | 7.24E-15 | *CD274,CD80,CD86,CXCL6,EGR1,IFI35,IFIT1,IFIT2,IFNA7,IFNG,IFNL2,IL18,IL21R,IL23A,LTA,NR4A1,OAS3,PYCARD,SERPINE1,TBX21,TLR7,TNC,TNF* |
| IRF3 | transcription regulator | -3.621 | 2.66E-11 | *CCRL2,CD274,CD58,CD70,CD86,CX3CL1,IFI27,IFIH1,IFIT1,IFIT2,IFNA7,IFNG,IFNL2,IL23A,OAS1,OAS3,TNF,TNFRSF4* |
| DDX58 | enzyme | -3.844 | 9.4E-27 | *C1S,CASP1,CCL26,CCL8,CD207,CD70,CTCFL,CXCL12,CXCL5,FEZ1,IFI27,IFI35,IFIH1,IFIT1,IFIT2,IFNL2,IL18,IL23A,IRAK2,NOD2,OAS1,OAS3,RELB,TBX21,THBS1,TLR5,TNF,TNFRSF18* |

**Figure S1:** Levels of different cell types in tumor specimens from good-NAC responders (GR) compared to poor-NAC responders (PR). ***, *p < 0.001*

**
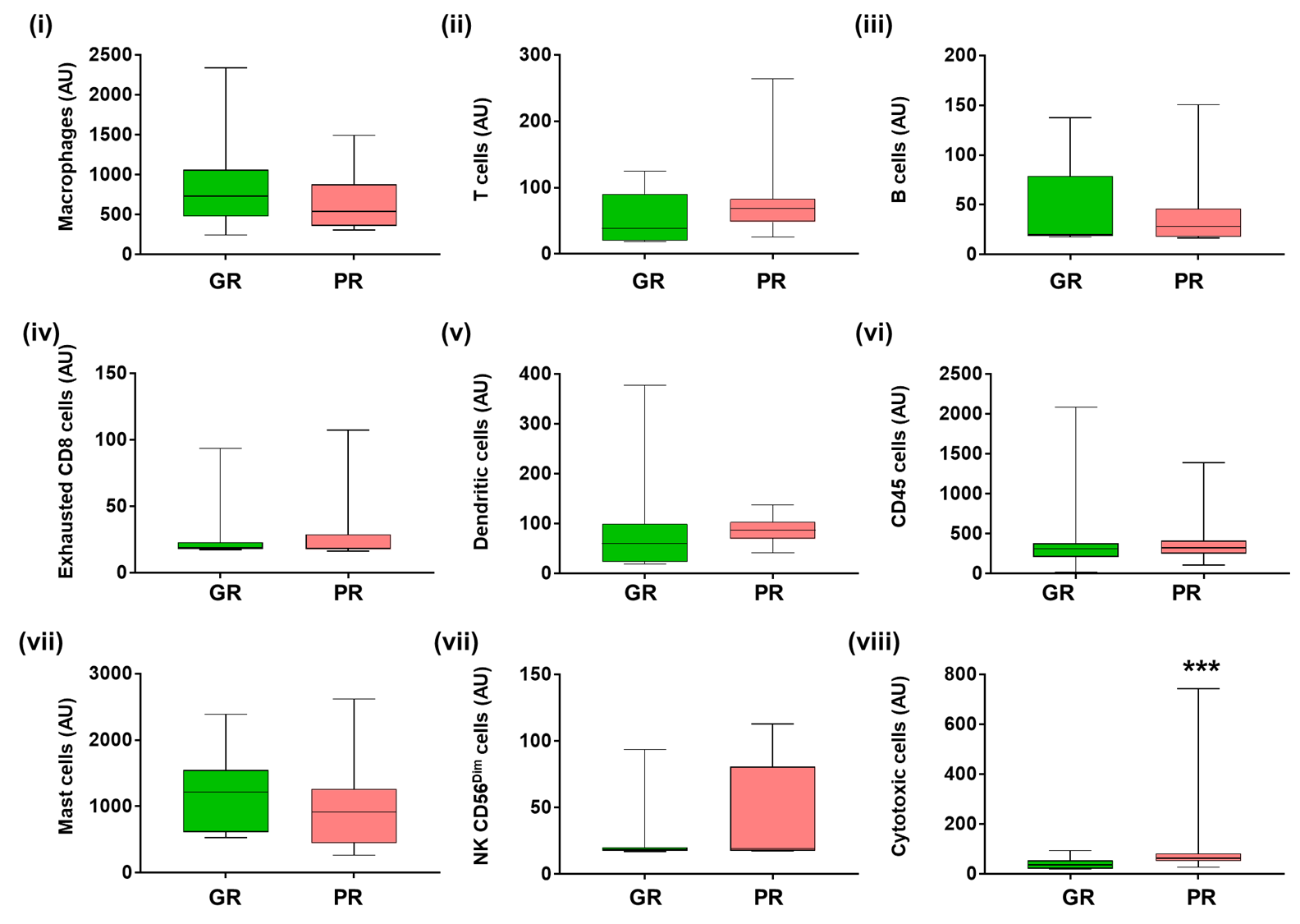
**
